# Advancements and challenges of nanorobots in surgical medicine: design, applications, and interdisciplinary integration

**DOI:** 10.3389/fbioe.2026.1778916

**Published:** 2026-03-24

**Authors:** Long Guo, Qing Lan, Fei Liu

**Affiliations:** 1 Department of Urology, The Second Affiliated Hospital of Nanchang University, Nanchang, China; 2 Department of Gastroenterology, The First Hospital of Nanchang, Nanchang, China

**Keywords:** biohybrid systems, minimally invasive surgery, nanomedicine, precision medicine, surgical nanorobots, targeted drug delivery

## Abstract

Surgical nanorobotics offers a strategy to surpass the physical constraints of conventional minimally invasive procedures. Designed to intervene at the cellular scale, these micro-agents facilitate high-precision tasks ranging from tumor margin delineation to targeted tissue manipulation. This article critically evaluates current actuation modalities—spanning magnetic, acoustic, optical, and chemical methods—for their efficacy in navigating complex physiological fluids. We specifically analyze the integration of propulsion systems with medical imaging, which is decisive for achieving the real-time feedback necessary for clinical safety in oncology and ophthalmology. Despite technical progress, widespread adoption faces persistent hurdles regarding long-term biocompatibility, deep-tissue control, and reproducible manufacturing standards. We conclude that bridging the gap between laboratory prototypes and clinical application requires shifting focus from novel locomotion schemes to comprehensive *in vivo* validation and standardized regulatory compliance.

## 1 Introduction

Nanorobots, positioned at the intersection of nanomedicine and surgical robotics, are transitioning from theoretical research to practical, verifiable systems designed for complex intraoperative tasks. Their core value lies in overcoming the limitations of macroscopic instruments, enabling precise operations at the microscopic level, where traditional tools cannot reach. Through active navigation, intelligent recognition, and on-demand execution, nanorobots perform critical tasks at cellular and subcellular levels, such as tumor boundary identification, local hemostasis, thermal therapy, chemotherapy drug delivery, minimally invasive removal, and closed-loop efficacy feedback (Soto et al., 2020; Sun et al., 2024; Chatterjee et al., 2024). Traditional surgery, limited by visibility and time constraints—particularly during tumor margin identification—often relies on frozen section pathology, which can result in a detection failure rate of up to 25%. However, the integration of Surface-Enhanced Raman Spectroscopy (SERS) technology, alongside other intraoperative sensing techniques, is redefining real-time molecular decision-making during surgery (Chen et al., 2025).

Despite these advancements, challenges remain for nanorobots, especially in areas such as stable propulsion within the body, real-time imaging of deep tissues, and precise remote control, particularly when confronted with the “last mile” technical bottleneck. Recently, an acoustically propelled absorbable hydrogel micro-robot (BAM) has successfully addressed these challenges by achieving stable propulsion, ultrasound visualization, and post-treatment biodegradation, paving the way from engineering development to clinical application (Han et al., 2024).

At the material and structural levels, DNA origami and nucleic acid logic gates enable molecular-level “computation-execution” coupling, overcoming the limitations of traditional two-state switches. This innovation allows nanorobots to handle multi-input, multi-level Boolean logic, controlling the sequence and spatial-temporal locations of operations (Pfeiffer et al., 2025; Abdollahzadeh et al., 2025; Asumadu et al., 2025). Regarding drive and control mechanisms, combinations of magnetic, acoustic, optical, electrical, and chemical drives, along with their hybrid versions, have been systematically evaluated. New paradigms have been proposed for compatibility with conductive or high-viscosity biological environments such as blood, mucus, and vitreous humor. For example, opto-electro-magnetic coupling nanorobots have achieved high-speed movement (>300 μm/s) in isosmotic conductive solutions and successfully completed biological cargo transport (Zhuang et al., 2025).

In terms of collective intelligence, magnetic heterogeneous micro-swarms can rapidly reconfigure between vortex and band-like shapes. Through division of labor, cooperation, and multi-point coordination with multi-level functional encoding, they can navigate complex environments and inhibit tumors via multiple pathways (Lu et al., 2024; Jin et al., 2021).

Substantial interdisciplinary progress is emerging. In oncology, the combination of the SERS-based intelligent nanoplatform with augmented reality (AR) navigation has created a closed-loop surgical process, ranging from preoperative tumor enrichment and molecular boundary warning during surgery to postoperative evaluation (Chen et al., 2025). In peritoneal metastasis treatment, a magnetic-laser integrated system drives the nanorobot swarm to accumulate against gravity, releasing chemotherapy drugs in a localized manner under ultrasound guidance with near-infrared (NIR) triggering (Wang et al., 2024). For spinal tumors, injectable nanorobot-hydrogel composites, triggered by NIR light, penetrate deeply to release thrombin, achieving preoperative embolization hemostasis while simultaneously using photothermal therapy to inhibit tumors, optimizing the balance between operative field safety and tumor control (Chen et al., 2024).

In vascular interventions, magnetic programmable colloidal micro-swarms can quickly detach wall-attached thrombi, magnetizing the thrombus debris into fragment robots for orderly recovery. This significantly shortens the reperfusion time during the critical 6-h stroke window and reduces the risks of distal re-thrombosis and prolonged exposure to nanomaterials (Wang et al., 2025).

Groundbreaking advances have also emerged in fields such as ophthalmic regeneration, maxillofacial surgery, endodontics, and neurointervention. For example, glucose-driven ultra-small nanorobots with single-atom coordination enhanced stem cell endocytosis and antioxidant microenvironment remodeling during corneal intervention (Ju et al., 2025). Titanium dioxide/silver (TiO2/Ag) light-driven nanorobots demonstrated effective propulsion in the visible light range and antibacterial effects during facial implant biofilm removal (Ussia et al., 2022). DNA nanorobots targeted post-transcriptional regulation of the 3′UTR gene in periodontitis, reprogramming fibroblasts for precise treatment (Zhang J. et al., 2024). In the central nervous system, a cross-scale “mother-child” system combining magnetic continuum robots with chemically/magnetically driven nanorobots achieved minimal intratumoral penetration for precise drug delivery after crossing the blood-brain barrier. Feasibility and preliminary safety were verified in *ex vivo* pig brain and animal models (Wu J. et al., 2024).

The application of nanorobots in surgical medicine represents a revolutionary approach to overcoming the major challenges faced in modern surgery. By addressing limitations in accuracy, microsurgical precision, and treatment implementation, these systems have the potential to improve surgical outcomes, enabling highly accurate, minimally invasive surgeries at the molecular and cellular levels. Integrating nanorobots into the surgical workflow will not only optimize surgical decision-making and enhance tumor margin identification accuracy but also facilitate the realization of targeted therapies, thus addressing critical challenges posed by traditional surgical methods.

However, despite the tremendous potential demonstrated by surgical nanorobots, their development still faces numerous challenges, with a significant gap between the current state and the expected goals. Major limitations include issues related to biocompatibility, control mechanisms, and the ability to navigate complex biological environments in real-time. Additionally, the lack of standardized manufacturing methods and difficulties in scaling these technologies for clinical applications present significant barriers to widespread adoption. In terms of clinical translation and governance, multiple reviews have emphasized the importance of establishing a comprehensive evidence chain from animal studies to clinical applications, along with standardized reporting and quality assessment systems. It has been noted that biodegradable materials, immune camouflage, imaging visibility, and closed-loop control will be crucial factors in advancing clinical applications (Soto et al., 2020; Sun et al., 2024; Agrahari et al., 2020; Soto and Chrostowski, 2018).

This paper aims to systematically review the latest advancements in surgical nanorobots, exploring their design principles, drive systems, and applications across various surgical fields. It also discusses the key obstacles that must be overcome in the translation of these technologies from preclinical research to clinical implementation.

To ensure a comprehensive and critical review, we conducted a systematic literature search using Web of Science, PubMed, and Google Scholar. The search window covered the period from 2015 to 2025, with a prioritized focus on breakthrough studies published in the last 3 years (2022–2025). Keywords included “surgical nanorobots,” “magnetic actuation,” “soft microrobotics,” “targeted drug delivery,” and “clinical translation.” The selection criteria prioritized peer-reviewed articles that demonstrated *in vivo* preclinical efficacy or novel interdisciplinary integration mechanisms.

## 2 Nanorobots in surgical medicine: definition, classification, and application scenarios

**FIGURE 1:**
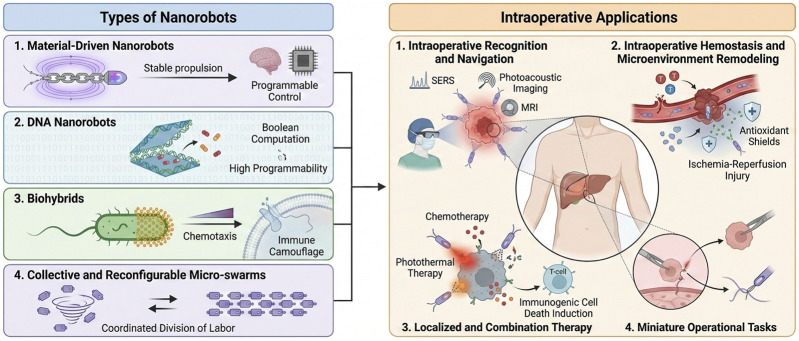
Classification and application scenarios of surgical nanorobots and four high-value intraoperative application scenarios.

In the medical field, nanorobots refer to miniature robots ranging from the nanoscale to the microscale. These robots can be passively or actively driven to perform preset tasks, including pure artificial material bodies, DNA/nucleic acid dynamic devices, biohybrids, and intelligent nanoplatforms (Soto et al., 2020; Sun et al., 2024; Zhou et al., 2021). Nanorobots can be categorized into four main types based on their design and functionality. The first category consists of material-driven artificial nanorobots, such as magnetic chain nanorobots, absorbable acoustic microrobots, and opto-electromagnetic coupled microrobots, which emphasize propulsion stability, programmable control, and imaging visualization (Han et al., 2024; Zhuang et al., 2025; Li et al., 2025). The second category includes DNA nanorobots, which utilize folding structures and nucleic acid logic to achieve molecular-level environmental sensing, Boolean computation, and on-demand release, offering high programmability and multi-input, multi-output capabilities (Pfeiffer et al., 2025; Abdollahzadeh et al., 2025; Asumadu et al., 2025; Wang M. et al., 2022). The third category encompasses biohybrids, which combine external membrane vesicles, sperm, bacteria, or cell membranes with inorganic materials, granting them chemotaxis, mobility, immune modulation, and immune camouflage properties (Tang et al., 2024; Zhang Y. et al., 2024; Li et al., 2018; Ding C. et al., 2023). The fourth category comprises collective and reconfigurable micro-swarms, which can switch between vortex and band-like forms through external fields and interaction encoding, achieving coordinated division of labor and closed-loop control (Lu et al., 2024; Jin et al., 2021; Zhou et al., 2020) (Figure 1).

Intraoperative constraints are strict, requiring system engineering trade-offs across multiple factors. In the complex *in vivo* environment, factors such as conductivity, turbulence, viscosity, and heterogeneous barriers necessitate the coupling of propulsion energy and material stability to meet stringent requirements. Clinical workflows rely on image guidance, closed-loop control, and real-time visualization. Furthermore, biological safety demands that nanorobots possess immune compatibility, biodegradability, metabolic clearance capacity, and low residual risk (Soto et al., 2020; Sun et al., 2024; Han et al., 2024). Thus, the design of surgical nanorobots must achieve a comprehensive balance across functionality, materials, imaging, and control, while collaborating effectively with macro-surgical systems, artificial intelligence planning, and surgeon interfaces to meet these complex intraoperative requirements.

### 2.1 Nanorobot design and driving platforms

DNA origami technology and nucleic acid computation provide nanorobots with a programmable molecular-level hardware foundation. Traditional two-state switches limit the scalability and temporal controllability of system responses. However, reconstructed DNA origami arrays, by coupling multiple two-state units into a network, enable nanorobots to integrate multiple operational modules, handle multi-input stimuli, execute multi-level Boolean logic, and precisely control operational sequences in the spatiotemporal dimension (Pfeiffer et al., 2025). In the field of tumor precision therapy, DNA origami not only carries oligonucleotide drugs for targeted release but also improves the manufacturability and operational efficiency of nanorobots through enzymatic amplification and the introduction of life-like artificial systems (Abdollahzadeh et al., 2025). Aptamer-based logic gates, such as AND, OR, NOT, and INHIBIT, enable molecular recognition, signal amplification, and cascade control in complex multi-input environments. The integration of the CRISPR-Cas system and multi-output gate cascades enhances the level of biosensing and targeted release. However, aptamer performance, cross-reactivity, and signal readout integration still face challenges (Asumadu et al., 2025) (Table 1).

**TABLE 1:** Nanorobot design, actuation, and coordination technologies.

Category	Specific Technology/Platform	Core mechanisms and advantages	Typical applications	Challenges and perspectives	References
Design and Driving Platforms	DNA Origami and Nucleic Acid Logic	High programmability: Multi-module integration through DNA network, multi-level Boolean logic execution and temporal control; Molecular computation and motion: Aptamer-based logic gates for molecular recognition; Autonomous DNA molecular motors achieve directional high-speed motion	Precision therapy: Targeted delivery of drugs/miRNA for tumor or periodontitis treatment; Intelligent response system: Adaptive anticoagulation regulation based on thrombin concentration; Nanomanufacturing: Seed platform for automated nano-synthesis	Aptamer performance, cross-reactivity, signal readout integration; Scalable manufacturing and *in vivo* stability	Pfeiffer et al. (2025), Abdollahzadeh et al. (2025), Asumadu et al. (2025), Zhang et al. (2024a), Siti et al. (2023), Liu et al. (2023), Yang et al. (2020)
	Multi-enzyme Cascade and Single-atom Engineering	Reaction-motion-therapy synergy: Multi-enzyme cascade reaction modules constructed using metal-polyphenol coordination; Endogenous fuel-driven: Enhanced diffusion and active motion through tandem catalysis using biological fuels like glucose	Tumor chemodynamic therapy: Glucose consumption, ROS generation, enhanced targeting and efficacy; Stem cell therapy assistance: Antioxidant and immune-regulating properties promote corneal regeneration	Ensuring enzyme stability and activity *in vivo*; Precise control of complex cascade reactions	Ju et al. (2025), Li et al. (2025)
Major Driving Mechanisms	Magnetic Drive	Fuel-free, remotely controllable, strong tissue penetration; Swarm reconfigurability: Magnetic micro-swarms can form vortex or band states and switch rapidly, adapting to complex environments	Targeted delivery and imaging-guided intervention; Swarm collaborative operations: Vascular embolization, biofilm removal, multi-point coordinated strikes	Precise control in complex dynamic environments; High requirements for external magnetic field equipment	Lu et al. (2024), Jin et al. (2021), Zhou et al. (2021), Zhang et al. (2025), Wang et al. (2022b)
	Acoustic Drive	Deep penetration, non-contact manipulation; Integrated driving and imaging: Serves as both propulsion energy source and ultrasound/photoacoustic imaging visualization tool; Biodegradable: Absorbable acoustic microrobots degrade via hydrolysis, reducing long-term risks	Long-duration stable propulsion with real-time ultrasound visualization; Closed-loop navigation: Combined photoacoustic/ultrasound dual-mode imaging for anatomical-functional feedback control	Maintaining propulsion efficiency in viscoelastic biological tissues; Achieving higher precision steering and manipulation	Han et al. (2024); Aziz et al. (2023); McNeill et al. (2020); Aziz et al. (2021)
	Light and Photothermal/Electrical Effect Drive	High-dimensional programmability: Polarization state enables single-particle orthogonal navigation with high control precision; Multi-physical field coupling: Thrust and torque generated through photocatalysis, thermophoresis, photoelectric effects	Microscale manipulation: Single-cell mechanics research, 3D imaging, micro/nanomachinery control	Limited light tissue penetration depth severely restricts deep *in vivo* applications	Zhan et al. (2019), Wang et al. (2018), Zheng et al. (2017), Di et al. (2022), Ding H. et al. (2023)
​	Electric Field and Electro-optical Coupling Drive	Multi-field synergistic enhancement: Improved propulsion efficiency in conductive biological fluids through structural-material synergy and multi-field coupling; Nanoscale manipulation: Programmable electrodynamic torque applied to highly charged DNA nanomechanical arms	Efficient biological cargo transport; Optimizing “electrically controlled” DNA nanomachine design	Limited propulsion efficiency in highly conductive biological fluids (e.g., blood)	Zhuang et al. (2025), Vogt et al. (2023)
	Chemical/Biological Fuel Drive	*In situ* energy supply with good tissue compatibility; Propulsion force generated through enzymatic reactions using endogenous substrates like hydrogen peroxide, glucose, and urea	Enhanced drug delivery: Improved membrane penetration and siRNA delivery efficiency; Specific environment applications: Irreplaceable in intracellular and specific organ environments	Difficult precise positioning control in complex *in vivo* environments	Tang et al. (2024), Schattling et al. (2017), Peng et al. (2023), Zhao et al. (2025)
Imaging and Collective Coordination	Imaging, Navigation and Human-Machine Interface (Enabling Technologies)	Multimodal imaging: Combined ultrasound/photoacoustic, SERS, MRI for multi-dimensional real-time tracking from anatomy to molecular level; Integrated “imaging-propulsion-control”: MRI navigation systems enable high-speed closed-loop control in deep tissues; Information enhancement and autonomous navigation: AR technology provides visual decision support for surgeons; AI algorithms enable autonomous path planning	Closed-loop navigation and controlled release; Real-time molecular warning of tumor boundaries; Autonomous intravascular navigation	Real-time fusion and registration of multimodal imaging information; Establishing efficient, low-latency control loops across macro-micro scales	Chen et al. (2025), Han et al. (2024), Aziz et al. (2023), Vartholomeos et al. (2011), Aziz et al. (2021), Martel (2007), Martel et al. (2008), Felfoul et al. (2008), Martel et al. (2009), Lu et al. (2019), Li et al. (2017), Chang et al. (2018)
	Collective Intelligence and Collaboration	Self-organization and reconfiguration: Magnetic swarms can self-organize into different morphologies, adapt to environments and execute complex tasks; Functional differentiation and collaboration: Multi-step, multi-path joint interventions through “division of labor-cooperation” mechanisms; Spontaneous synchronization: Chemical coupling enables self-synchronization without external complex control	Complex task execution: Navigating tortuous narrow environments, multi-pathway coordinated strikes on tumors; Molecular-scale collective operations: Extending swarm experience to molecular level for higher-density parallel operations	Requires robust collective closed-loop feedback, pattern encoding and collaborative scheduling strategies	Lu et al. (2024), Jin et al. (2021), Zhou et al. (2020), Wa et al. (2024)

Nucleic acid-based orbital motion devices embed computation-execution functionality within their kinematics. Non-bridge-burning autonomous DNA molecular motors, operating on the origami platform, achieve directional and high-speed translation through self-closed loop orbits, capable of completing complex displacements via sharp turns. These serve as seed platforms for scalable automated nanomanufacturing or synthesis (Siti et al., 2023). Light-driven DNA walkers, in combination with long-rod origami structures, achieve reversible and repeatable displacement under pure mechanical drive, demonstrating their portability in the field of light-controlled nanorobots (Liu et al., 2023). Additionally, nucleic acid logic can be coupled with functional payloads, such as coagulants, anticancer small molecules, and miRNAs, to form intelligent release systems. For example, DNA nanorobots in thrombus environments can trigger adaptive anticoagulant responses based on thrombin concentration thresholds, regulating and preventing bleeding risks (Yang et al., 2020). In periodontitis treatment, DNA nanorobots targeting pathological fibroblasts’ 3′UTR gene post-transcriptionally regulate and deliver miR-1-3p, precisely inhibiting pathogenic gene expression, significantly reducing inflammation, and promoting regeneration (Zhang J. et al., 2024).

In addition to nucleic acid platforms, programmable multi-enzyme cascades, metal-polyphenol coordination, and single-atom site engineering extend the synergy of reaction-motion-therapy. Linear chain magnetic nanorobots, fixed with metal-polyphenol coordination, enhance peroxidase-like activity. By assembling glucose oxidase, copper ions (Cu), and human serum albumin (HSA) on the surface, they construct multi-enzyme cascade reaction modules, forming an integrated system for tumor chemodynamic therapy. This system consumes glucose, generates reactive oxygen species (•OH), and depletes glutathione (GSH), enhancing *in vivo* targeting and therapeutic effects (Li et al., 2025). Single-atom CeO2-Au nanorobots, powered by glucose, achieve enhanced diffusion and active motion through tandem catalysis. They possess intrinsic antioxidant and immune-regulating properties, facilitating stem cell therapy to promote corneal regeneration (Ju et al., 2025).

The critical analysis presented in Table 1 reveals a fundamental trade-off between propulsion autonomy and control precision within current actuation strategies. A central insight emerging from this synthesis is that no single platform or propulsion modality is universally superior; rather, the efficacy of each is contingent upon specific surgical requirements and the biological environment. External field-driven systems (magnetic and acoustic) offer superior controllability and biocompatibility, rendering them the preferred modality for precision interventions such as microsurgery and targeted ablation. However, their reliance on bulky external infrastructure restricts their applicability in deep-seated or anatomically complex regions susceptible to field gradient attenuation. Conversely, chemical and biological motors exhibit high autonomy and energy conversion efficiency but face significant challenges regarding fuel toxicity and immune clearance. Consequently, the future of surgical nanorobotics likely lies in intelligent hybrid systems that integrate the complementary strengths of these technologies to circumvent the limitations of single-mode actuation within complex physiological environments.

### 2.2 Main driving mechanisms and applications of nanorobots

Magnetic drive has become one of the leading propulsion methods for *in vivo* nanorobots due to its fuel-free nature, remote programmability, and tissue penetration capabilities. Magnetic nanorobots can achieve helical or flexible propulsion, or surface walking, through rotating magnetic fields. The magnetic chain structure enhances magnetic responsiveness and simplifies design, making it ideal for targeted delivery, imaging-guided interventions, and surgical assistance (Zhou et al., 2021; Zhang et al., 2025; Wang Z. et al., 2022). Magnetic micro-swarms, through external field temporal encoding and particle interactions, can form various shapes such as vortices or bands, quickly switching between them. This enables traversal of complex environments and multi-point cooperative work with closed-loop control (Lu et al., 2024; Jin et al., 2021) (Table 1). Magnetic fields offer precise control. However, generating the strong gradients required for deep-tissue manipulation necessitates exponentially higher power consumption and massive coil configurations. Consequently, this scaling challenge currently restricts the integration of magnetic actuation systems into standard clinical operating suites.

Acoustic drive offers unique advantages in deep penetration and non-contact manipulation, serving both as a propulsion energy source and a visualization tool. Cavity-type acoustic microrobots achieve long-distance self-propulsion through resonant cavity design. Recently, they have achieved wafer-scale fabrication (>10^9), size control from 500 nm to 5 μm, and three-dimensional autonomous motion capabilities (McNeill et al., 2020). Absorbable acoustic microrobots, with a dual-opening bubble cavity design and hydrophilic/hydrophobic surface chemical optimization, enable stable propulsion for several days with real-time ultrasound visualization. After treatment, they degrade via hydrolysis, reducing long-term risks (Han et al., 2024). Photoacoustic/ultrasound imaging technology provides dual-mode feedback for anatomical and functional tracking at deep *in vivo* levels, supporting closed-loop navigation (Aziz et al., 2023; Aziz et al., 2021). While acoustic propulsion enables deep tissue penetration, its efficiency is severely compromised in air-filled organs, such as the lungs and intestines. This is caused by significant acoustic impedance mismatch. Furthermore, without precise regulation, high-intensity focused ultrasound poses safety risks. These include inadvertent thermal injury and cavitation-induced damage to surrounding tissues (Zhao et al., 2025).

Light and photothermal/electro effects provide photodriven nanorobots with higher-dimensional programmability. Visible and near-infrared light can generate thrust and torque through photocatalysis, electrophoresis, thermophoresis, and photoelectric effects. Polarization-state encoding allows for orthogonal navigation of individual particles, significantly reducing light intensity (Zhan et al., 2019; Wang et al., 2018; Zheng et al., 2017). A universal photo-thermal-electric coupling rotational platform can drive symmetrical particles to rotate out of plane at low-power laser distances, demonstrating good compatibility in biological environments (Di et al., 2022). By utilizing multiple thermal-induced phenomena such as photothermal-thermoelectric-thermophoretic effects, photothermal rotation and manipulation technology provides general modules for single-cell mechanics, 3D imaging, and micro/nanomachinery control (Ding H. et al., 2023).

Electric fields and electro-optic coupling technologies face certain limitations for propulsion in conductive biological fluids, but through structural-material synergy and multi-field coupling, propulsion efficiency can be significantly enhanced. For example, electro-optic coupled α-Fe2O3@aTiO2/Au flower-like composite microrobots in isosmotic conductive glucose solutions achieve efficient propulsion through enhanced photocatalysis and surface structures, while also using electric fields and uniform external magnetic fields to reach speeds exceeding 300 μm/s. These microrobots can transport various biological cargos (Zhuang et al., 2025). At the nanoscale, electric fields can apply programmable electro-dynamic torque to highly charged DNA nanomechanical arms. Single-molecule fluctuation analysis reveals electro-hydrodynamic coupling principles, optimizing the design of “electro-controlled” DNA nanomachines (Vogt et al., 2023). However, the clinical translation of these strategies faces inherent physical barriers. Light penetration depth in biological tissues is limited. This restricts the application of light-driven systems in deep-seated regions. Furthermore, the associated photothermal effect poses a risk of collateral thermal damage to surrounding healthy cells.

Similarly, electrically driven systems encounter challenges in physiological environments. The high ionic strength of body fluids induces the Debye screening effect. This phenomenon significantly attenuates the effective electric field, thereby compromising *in vivo* propulsion efficiency (Zhao et al., 2025).

Chemical and biofuel propulsion strategies prioritize *in situ* energy harvesting and tissue biocompatibility. Endogenous substrates such as hydrogen peroxide, glucose, and urea can be enzymatically catalyzed to generate concentration gradients for propulsion. For instance, dual-engine systems—such as those pairing glucose oxidase-functionalized platinum nanoparticles with trypsin—exhibit enhanced diffusivity through dual-fuel synergy while enabling directional navigation via magnetic response (Schattling et al., 2017). Similarly, outer membrane vesicle-urease biohybrids demonstrate self-propulsion within urea-rich environments, facilitating transmembrane crossing and siRNA delivery (Tang et al., 2024).

Recent advances have accelerated the preclinical translation of these technologies. Simó et al. reported that radionuclide-labeled urease nanorobots could actively penetrate the mucosal barrier in an orthotopic bladder cancer mouse model, resulting in a significant 90% reduction in tumor volume (Simó et al., 2024). Additionally, a tri-enzyme cascade system incorporating glucose oxidase, catalase, and urease (GOx-CAT-URE) utilized multi-substrate synergy to achieve deep tumor penetration (>0.55 mm) and effective starvation therapy (Gao et al., 2025). Furthermore, neutrophil-based biohybrid motors have been shown to autonomously traverse the blood-brain barrier (BBB) via chemotaxis along inflammatory gradients, effectively targeting and clearing residual post-surgical glioma cells (Li et al., 2026).

Current perspectives indicate that while chemical propulsion faces challenges regarding precise positional control in complex *in vivo* environments—areas where magnetic and ultrasound propulsion show greater translational promise—it retains unique advantages for intracellular and organ-specific targeting (Wang and Zhou, 2021; Peng et al., 2023). Specifically, the superior microenvironmental adaptability and biosafety of enzyme-powered systems represent a critical breakthrough for current clinical translation efforts (Zhao et al., 2025). However, chemically driven motors face the challenge of fuel depletion over time. Furthermore, the accumulation of metabolic byproducts—such as ammonia from urease activity—may induce localized toxicity. Consequently, rigorous biocompatibility assessments are essential for long-term therapies (Zhao et al., 2025).

### 2.3 Nanorobot imaging and collective coordination

Imaging visualization and positioning navigation are fundamental to intraoperative applications. Ultrasound and photoacoustic imaging provide nanorobots with high spatiotemporal resolution tracking capabilities. Ultrasound offers real-time and penetration advantages, while photoacoustic imaging enhances contrast through molecular-specific absorption. Together, these technologies enable dual-mode anatomical-molecular closed-loop control, successfully demonstrated for real-time controllable navigation and payload release in mouse uterine and bladder models (Aziz et al., 2023; Aziz et al., 2021). Acoustic microrobots, through cavity oscillation-induced enhancement of ultrasound scattering, incorporate contrast functions that improve the precision of *in vivo* real-time tracking (Han et al., 2024). SERS combines single-molecule fingerprints with plasmonic enhancement to create self-navigation/stimulus-responsive signal amplification probes. These probes can delineate tumor boundaries and provide molecular warnings in real-time during surgery. When integrated with AR navigation systems, they form a closed-loop workflow, spanning from preoperative enrichment to postoperative validation (Chen et al., 2025) (Table 1).

Magnetic Resonance Imaging (MRI)-guided and MRI navigation systems provide an integrated “imaging-propulsion-control” ability for magnetic devices at deep *in vivo* levels. MRI gradient-based propulsion, tracking, and closed-loop control technology has achieved 24 Hz closed-loop control and 10 cm/s navigation speeds in living arteries, demonstrating the manipulation potential of nanomicro devices for clinical applications (Martel, 2007; Martel et al., 2008). MR tracking technology selectively excites magnetic signatures to achieve three-dimensional localization on single-axis k-space projections, confirming feasibility in in vivo tortuous blood vessels and animal models (Felfoul et al., 2008). MRI navigation platform architectures utilize nanovesicles, bacteria, or magnetic particles as wireless mechanical arms, enabling precise targeting of deep lesions in microvascular networks following predetermined trajectories. This forms an integrated “imaging-propulsion-feedback” system (Vartholomeos et al., 2011; Martel et al., 2009).

AR and human-machine interface technologies establish an information and control channel between the surgeon and the nanorobots. By overlaying AR with SERS/photoacoustic multimodal imaging, molecular boundaries are visually presented, aiding intraoperative decision-making (Chen et al., 2025). Micro-manipulators based on acoustically enhanced local flow can map frequencies and durations to user commands, achieving large-scale parallel transportation of particles/cells under human-machine interfaces based on keyboard typing or audio signals (Lu et al., 2019). In macro-micro cross-scale cooperation, Artificial Intelligence (AI) path planning and visual closed-loop control technologies enable autonomous navigation of microrobots in complex obstacles and dynamic environments, demonstrating a fully automated closed-loop system prototype consisting of “microscopic-AI-magnetic field generator (Li et al., 2017)”. The A* shortest path on MRA images provides automated path solutions for intravascular magnetic navigation (Chang et al., 2018).

Faced with complex surgical tasks, the limitations of individual performance can be overcome through collective intelligence and collaboration mechanisms. Magnetic micro-swarms can self-organize into vortex or band states under programmable magnetic fields and quickly switch between these states, adapting to navigate tortuous branches and narrow environments. By assigning specialized functions to different subgroups, multi-step joint interventions such as “division of labor-cooperation-multi-path strikes” can be achieved. For example, using a domino effect to disrupt multiple growth pathways of tumors results in over 90% success in reaching complex environments with significant tumor suppression (Lu et al., 2024). Reviews indicate that group control requires closed-loop feedback, pattern coding, and collaborative scheduling of inter-group interactions. Magnetic swarms have shown potential clinical value in image-guided delivery, vascular embolization, and biofilm removal applications (Jin et al., 2021).

Chemical coupling and acoustic/electrical field assistance provide new means for self-organization and synchronization. Oscillating Ag Janus micromotors spontaneously synchronize due to the coupling of substrates and flow fields when in proximity, with collective beating frequency primarily driven by electroosmosis at the substrate. This reveals a biological-mimetic self-coordination mechanism that does not require external complex control (Zhou et al., 2020). At the molecular scale, collective molecular machines achieve shape deformation and motion amplification through space-time integration, providing theoretical and design guidelines for “collective molecular devices-reconfigurable materials-nano operations.” This inspires the transformation of micro/nanorobot swarm experiences into the molecular level, achieving higher-density parallel operations (Wa et al., 2024).

## 3 The prospects and challenges of nanorobots in surgical applications

### 3.1 Tumor surgery and localized treatment

**FIGURE 2:**
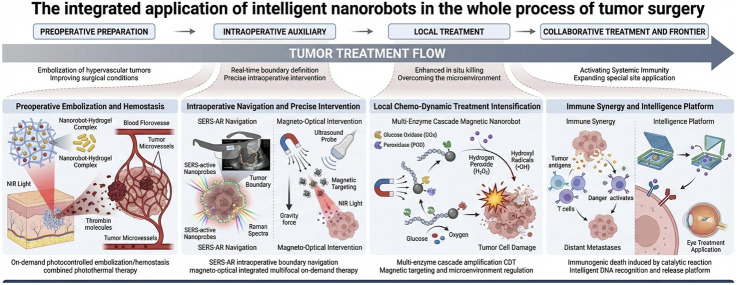
Main applications of nanorobotics in cancer therapy.

The application of nanorobots in tumor surgery centers on achieving precise intraoperative identification, navigation, and highly efficient localized treatment. Regarding identification and navigation, these systems leverage the fusion of multimodal imaging technologies—including SERS, photoacoustic imaging, ultrasound, and MRI—with AR navigation systems. This integration aims to address critical challenges associated with uncertainty in tumor margin delineation and delays in intraoperative decision-making (Chen et al., 2025; Aziz et al., 2023; Vartholomeos et al., 2011). Tumor surgery faces challenges such as uncertainty in margin determination and long intraoperative decision-making times, which can affect both the efficacy and efficiency of the surgery. SERS-active intelligent nanoplatforms offer molecular-morphology integrated intraoperative boundary identification. Signal amplification probes and self-navigating nanorobots enable accumulation and real-time warnings within the tumor microenvironment. When combined with AR navigation systems, they form a closed-loop workflow that significantly shortens decision delays and reduces the risk of incomplete tumor resection (Chen et al., 2025). Regarding localized therapy, nanorobotic systems have enabled the implementation of diverse synergistic therapeutic strategies. In peritoneal metastasis models, a magnetic-laser integrated system drives DOX-loaded magnetic nanorobot swarms to accumulate against gravity in the peritoneal cavity. Ultrasound-guided localization triggers localized photothermal drug release using near-infrared (NIR) light, enabling multi-focal on-demand treatment while reducing off-target thermal damage (Wang et al., 2024). For tumors with abundant blood supply, preoperative arterial embolization or hemostatic treatments can significantly improve resection conditions. Injected nanorobot-silk fibroin hydrogel complexes loaded with thrombin use NIR to penetrate deep into the tumor and release thrombin on demand, embolizing microvessels while also combining with gold nanorods for photothermal tumor suppression. This approach significantly reduces intraoperative bleeding and postoperative recurrence, showing promising preoperative minimally invasive results in a mouse vertebral metastasis model (Chen et al., 2024) (Figure 2). However, significant physiological barriers hinder clinical translation. Beyond the rapid clearance by the reticuloendothelial system (RES) which reduces bioavailability, the dense extracellular matrix and high interstitial fluid pressure within solid tumors severely restrict the deep penetration of nanorobots, often limiting therapeutic effects to the tumor periphery. Furthermore, regarding the aforementioned inorganic agents their long-term non-biodegradability raises concerns about chronic toxicity and potential interference with future diagnostic imaging, necessitating rigorous longitudinal safety evaluations (Chen et al., 2024; Zhao et al., 2025).

In the localized treatment of solid tumors, multi-enzyme cascade magnetic nanorobots generate reactive oxygen species (H2O2) and peroxidase-like activity via glucose oxidase (GOx) within the tumor, producing hydroxyl (•OH) free radicals. They also deplete glutathione (GSH) with copper ions, significantly enhancing chemodynamic therapeutic effects. Additionally, an external magnetic field promotes their *in vivo* targeted accumulation (Li et al., 2025). Magnetic nanorobots combined with immune linkage strategies have demonstrated a 59.3% inhibition rate in breast cancer treatment, suggesting the potential of coupling chemical catalytic reactions to induce immunogenic cell death and activate systemic anti-tumor immune responses (Guo et al., 2023). DNA nanorobots provide an intelligent recognition-on-demand release paradigm for tumor treatment. DNA origami and nucleic acid computation platforms have advantages in targeted oligonucleotide drug delivery. Reviews indicate that enzyme amplification and process simplification can enhance large-scale production, while combining nucleic acid circuits and machine learning offers new possibilities for personalized tumor precision therapy (Abdollahzadeh et al., 2025). In the treatment of ocular tumors and diseases related to angiogenesis, vitreous injection has become a key method for intraoperative and perioperative treatment. Intelligent-responsive delivery and nanorobots are considered the next-generation approach for achieving neurovascular active regulation (Lin et al., 2025).

### 3.2 Vascular intervention and thrombosis treatment

**FIGURE 3:**
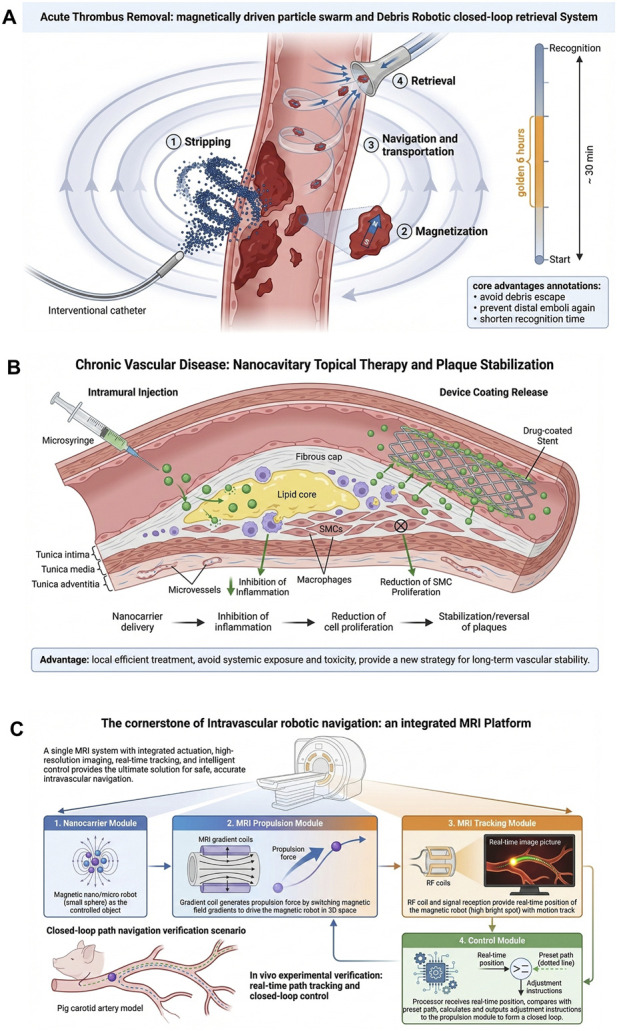
Application of nanorobots in vascular intervention. **(A)** A programmable magnetic colloidal micro-swarm system for acute thrombectomy, demonstrating the process of thrombus detachment, magnetization of debris, and recovery. **(B)** Nanotechnology strategies for atherosclerosis, showing localized delivery to inhibit plaque inflammation and stabilize vessel walls. **(C)** MRI-based navigation system integrating imaging, propulsion, and closed-loop control for guiding magnetic microdevices in deep vascular networks.

In the realm of vascular intervention, beyond thrombus clearance, nanorobots demonstrate significant potential for intraoperative hemostasis and modulation of the vascular microenvironment—for instance, through the targeted delivery of thrombin for precise hemostasis or the mitigation of ischemia-reperfusion injury (Chen et al., 2024; Ding C. et al., 2023). Nevertheless, the recanalization of acute thrombosis remains one of the most pressing clinical challenges, characterized by a highly sensitive balance between time-dependent benefits and procedural risks. Wang et al. developed a programmable colloidal micro-swarm that is placed inside the blood vessel via a catheter. By using an external magnetic field, the micro-swarm rapidly detaches wall-attached thrombi, after which the thrombus fragments are magnetized into fragment robots. These robots are propelled along a predetermined helical trajectory to a controllable location and then recovered using catheter suction. This process—from thrombus detachment and fragment magnetization to propulsion, recovery, and reperfusion execution—can be completed in approximately half an hour. It significantly shortens the time window for reperfusion within the critical “golden 6 h” of stroke and reduces the risk of distal re-thrombosis and prolonged exposure to circulating nanoparticles in the blood (Wang et al., 2025) (Figure 3A). Additionally, the study highlights that the propulsion path of the magnetized fragment swarm, its magnetic response characteristics, and the method of fragment recovery are key design dimensions for achieving a safe, controllable, and rapid thrombosis removal system.

In chronic vascular diseases such as atherosclerosis, nanotechnology also shows significant potential in vascular interventional therapy. Zong et al.’s review article notes that nanocarrier systems and localized delivery strategies are being utilized to inhibit inflammation within plaques and endothelial cell proliferation, thereby stabilizing or reversing plaque progression (Zong et al., 2024) (Figure 3B). The review emphasizes that injecting nanoparticles into the vessel wall or coating interventional devices with nanomaterials allows for efficient localized release, avoiding systemic drug exposure and toxicity. This nanotechnology toolbox provides new strategies for vascular surgery, with applications ranging from disease prevention to intraoperative assistance. Therefore, aside from the thrombectomy process, nanomaterial-assisted strategies for vascular structural reconstruction and long-term stability should not be overlooked.

“Imaging-navigation-execution” integrated technology plays a key role in controlling the propulsion of vascular robots. Vartholomeos et al.’s early review systematically examined the use of MRI systems as a propulsion drive, imaging tracking, and closed-loop control platform for nanorobotic and microrobotic systems. The proposed four-module system (nanocarrier, MRI propulsion module, MRI tracking module, and control module) laid the theoretical foundation for subsequent vascular navigation research. Meanwhile, Martel et al. validated the real-time control and path navigation of untrailed magnetic microspheres using a clinical MRI system in a pig carotid artery model. This enabled pre-planned path tracking and closed-loop control within the blood vessels, providing pioneering data for the feasibility of magnetic microdevices in real vascular navigation (Vartholomeos et al., 2011; Martel et al., 2008) (Figure 3C). Despite these advances, intravascular manipulation carries inherent risks. A critical limitation is the hemodynamic instability of nanorobot swarms; high shear stress in major arteries can disrupt swarm cohesion, potentially leading to unintended embolization in distal capillaries. Moreover, achieving real-time, high-resolution tracking in fast-flowing blood remains a bottleneck, as current MRI or ultrasound feedback often suffers from latency, compromising safety during critical interventional procedures (Wang et al., 2025; Vartholomeos et al., 2011; Martel et al., 2008).

### 3.3 Minimally invasive gastrointestinal and organ dysfunction treatment

The gastrointestinal environment presents extreme challenges for nanorobots due to its high viscosity, significant pH gradients, abundant enzymatic activity, and complex fluid dynamics, such as peristalsis, secretion fluid flow, luminal twists, and mucosal fluctuations. A review in digestive surgery highlights that this complex environment forces nanorobots to evolve from traditional “static components” to “smart entities” with mobility, responsiveness, feedback, and autonomous navigation capabilities. Specifically, the design of nanorobots must be optimized in terms of propulsion mechanisms, material resistance to enzymatic degradation, mucosal penetration ability, and intraoperative closed-loop feedback control. Organ-specific challenges, such as gastrointestinal peristaltic displacement, concentration gradient switching, mucus barriers, and pH changes, must also be addressed. The integration of artificial intelligence (AI) platforms and overcoming organ-specific issues is accelerating the development of this technology, bringing it closer to human trials. At the same time, ethical, legal, and clinical translation preparations must progress alongside technological advancements to ensure safety and effectiveness (Rui et al., 2025).

**FIGURE 4:**
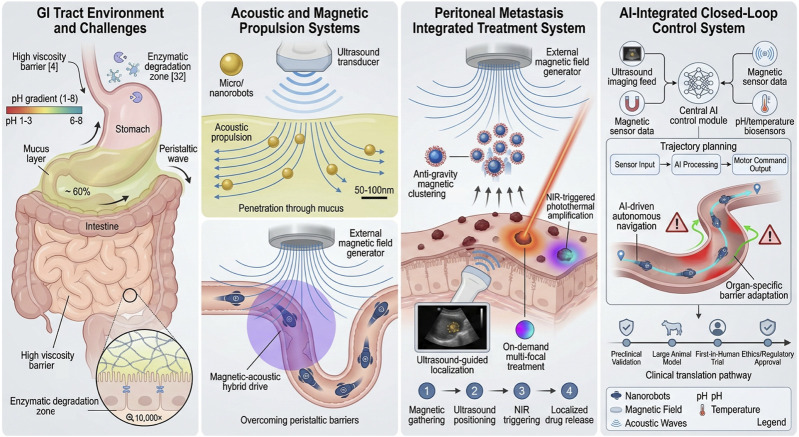
Application of nanorobots in the GI tract.

In terms of propulsion mechanisms, acoustic propulsion and sound capture technologies have become core methods for micro/nanorobot applications in the gastrointestinal cavity due to their strong penetration and remote, non-contact manipulation capabilities. Studies indicate that acoustic waves can achieve traction, suspension, and propulsion of robots in high-viscosity or complex fluid environments, while ultrasound imaging technology provides real-time monitoring (Cao et al., 2024). The advantages of acoustic propulsion are particularly evident in high-viscosity environments, where it can effectively overcome fluidic barriers. Additionally, magnetic-acoustic hybrid drive technology (combining magnetic field guidance and acoustic wave propulsion) has shown great synergistic potential in intraluminal propulsion, particularly for navigating bends, fluctuating fluid environments, and mucus-attached areas in the gastrointestinal tract (Wu G. et al., 2024) (Figure 4).

In organ cavity treatments, the integrated application of magnetic nanorobot systems has demonstrated breakthrough potential. Research has shown that a laser-magnetic integrated system, applied in peritoneal metastasis treatment, can control the reverse gravity accumulation of nanorobots within the peritoneal cavity using an external magnetic field. Local thermal effects triggered by laser guidance induce drug release, achieving a “cavity-point alignment-local heat-drug synergistic treatment” model. The feasibility of this system within the peritoneal cavity has been experimentally validated, demonstrating precise directional control and effective therapeutic outcomes during the treatment process (Wang et al., 2024). Nevertheless, the complex biochemical environment of the GI tract poses a hurdle for long-term functionality. Non-specific adsorption of biomacromolecules (bio-fouling) on nanorobot surfaces can rapidly degrade sensor accuracy and propulsion efficiency. Additionally, ensuring the complete excretion of non-degradable microrobots after the task is vital to prevent potential mucosal irritation or gastrointestinal obstruction, particularly in patients with compromised motility (Rui et al., 2025).

### 3.4 Ocular, maxillofacial, and dental treatments

**FIGURE 5:**
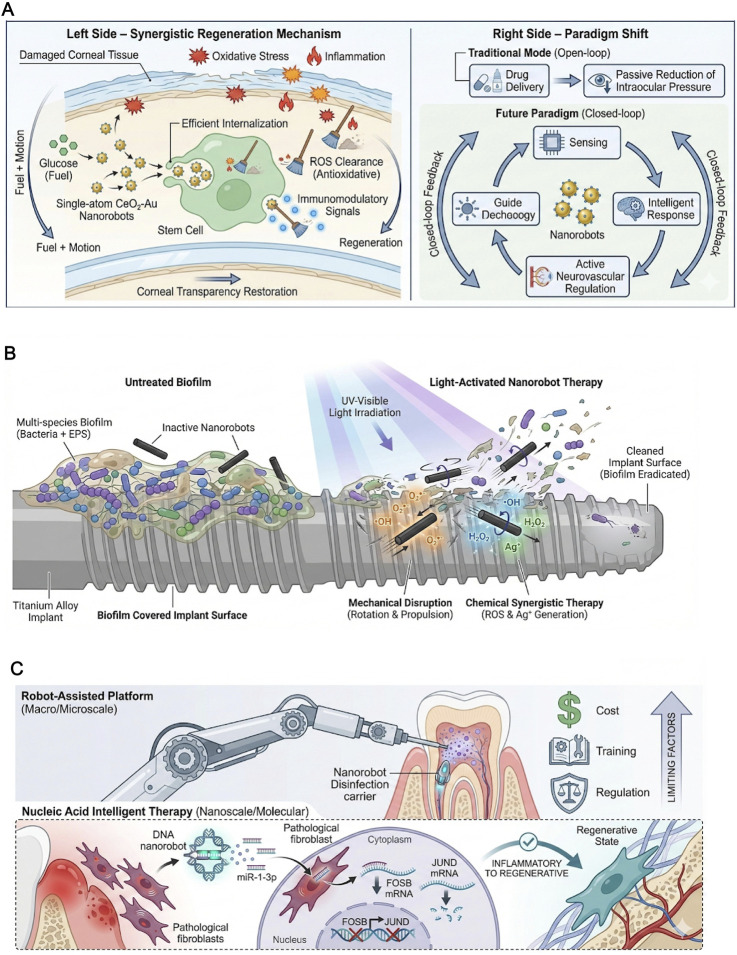
Nanorobots in Ocular, Maxillofacial, and Dental Treatments. **(A)** Single-atom CeO_2_-Au nanorobots for corneal regeneration, promoting stem cell endocytosis and antioxidant microenvironment remodeling. **(B)** Light-driven TiO_2_/Ag nanorobots for the mechanical and chemical removal of multi-species biofilms on facial titanium implants. **(C)** DNA nanorobots targeting the 3′UTR region for gene post-transcriptional regulation in periodontitis fibroblasts to reduce inflammation and promote tissue repair.

Corneal regeneration and intraocular treatments have long been hindered by biological barriers, as well as the adverse effects of postoperative oxidative stress and inflammatory microenvironments. Recent studies have reported the application of single-atom CeO_2_-Au nanorobots. This system uses glucose as fuel and improves the efficiency of stem cell endocytosis by approximately twofold through catalytic mechanisms, while also providing intrinsic antioxidant and immune-regulatory functions. These capabilities help reshape the microenvironment of the damaged cornea. In a mouse alkali-burned cornea model, this method significantly accelerated the recovery of corneal transparency, demonstrating the effectiveness of the “energy–motion–microenvironment” synergistic design (Ju et al., 2025) (Figure 5A). Furthermore, in managing intraocular diseases, particularly conditions like glaucoma, vitreous injection has become the mainstream treatment modality for intraoperative and perioperative care. This approach overcomes poor compliance with eye drops and the high complication rate of filtering surgeries. A recent review highlighted that future treatments will no longer be limited to passive intraocular pressure reduction but will shift toward active neurovascular regulation. Intelligent-responsive systems and nanorobots are considered key technologies for this transformation, emphasizing a shift from simple drug delivery to a “perception–response–control” closed-loop system (Lin et al., 2025).

Maxillofacial implants and the associated challenges of infection and biofilm formation are significant concerns in surgical complication management. Multi-species biofilms are commonly found on the surface of titanium alloy facial bone plates. Studies have shown that light-driven black TiO_2_/Ag tubular nanorobots can achieve efficient propulsion and rapid rotational motion in the ultraviolet-visible light range. In a model of multi-species biofilm on facial titanium plates, they significantly reduced the biofilm load. This suggests a novel strategy for controlling perioperative implant infections—mechanical disruption of persistent biofilms by nanorobots combined with light-chemical synergistic therapy (Ussia et al., 2022) (Figure 5B). In dental pulp treatment, robotic systems, including robotic arms, AI navigation, and nanorobotic disinfection carriers, show potential in improving treatment precision, reducing operator fatigue, and enhancing minimally invasive effects. A recent review indicated that robotic-assisted root canal treatments and robotic systems in pulp therapy are on the rise, although widespread adoption is still hindered by issues such as cost, training, and regulation (Adil et al., 2025). Meanwhile, DNA nanorobots based on nucleic acid engineering have achieved targeted gene post-transcriptional reprogramming in periodontal treatment. For example, by targeting the 3′UTR region of periodontal pathogenic fibroblasts and delivering miR-1-3p to downregulate FOSB and JUND gene expression, inflammation was reduced, and tissue regeneration was promoted. In animal models, this system showed excellent anti-inflammatory and reparative effects (Zhang J. et al., 2024) (Figure 5C). However, clinical application in these sensitive regions faces unique challenges. For ocular treatments, the delicate balance between retention and clearance is critical; prolonged retention of metallic-based nanorobots in the vitreous humor may induce opacity or retinal toxicity. In dental and maxillofacial contexts, the resilience of the multi-species oral microbiome often leads to rapid re-colonization, challenging the long-term efficacy of single-use nanorobotic disinfection strategies (Wang et al., 2025; Lin et al., 2025).

### 3.5 Neurosurgical and orthopedic applications

**FIGURE 6:**
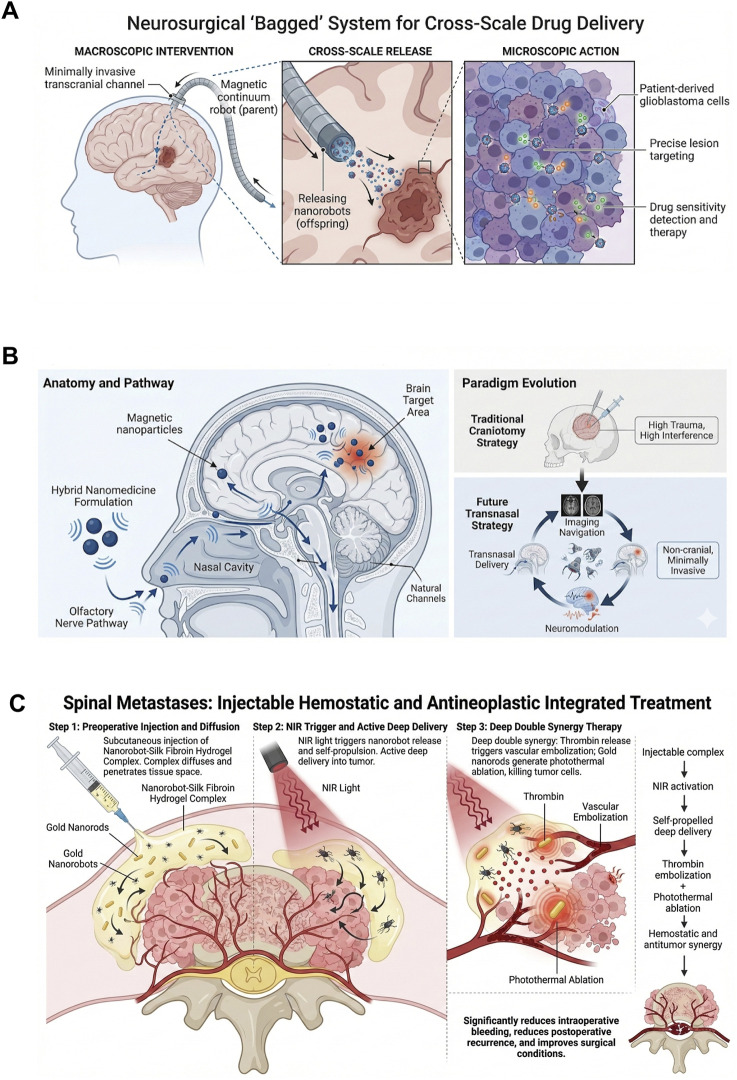
Nanorobotic Strategies in Neurosurgery and Orthopedics. **(A)** The cross-scale “Marsupial” robotic system (combining a magnetic continuum robot and nanorobots) for precise drug delivery across the blood-brain barrier. **(B)** Schematic of the intranasal delivery route leveraging hybrid nanomedicine for central nervous system access. **(C)** Mechanism of the “Hemostasis-Antitumor Integration” using nanorobot-hydrogel composites for treating spinal metastases.

Neurosurgery and orthopedic surgery demand exceptional operative precision, driving the evolution of nanorobotics toward micromanipulation at the tissue and cellular levels—including micro-scale suturing, grasping, and cutting (Jiang et al., 2025; Zhou et al., 2024; Shang et al., 2016; Yang et al., 2015). However, a critical challenge in these high-precision interventions remains the effective traversal of biological barriers for targeted drug delivery. Specifically, delivery to the central nervous system is significantly impeded by the blood-brain barrier (BBB) and the challenges of achieving high-resolution spatial localization within complex anatomical structures. In response, the cross-scale “marsupial” system has been proposed. This system utilizes a micro-magnetic continuum robot (“mother”) as the primary instrument, which enters the cranium via a minimally invasive transcranial pathway (such as the Ommaya infusion reservoir). Once inside, it releases a chemical/magnetic hybrid nanorobot directly to the lesion site, transitioning from macroscopic targeting to microscopic fine localization. In the study, drug sensitivity testing was performed using patient-derived glioblastoma cells *in vitro*, and the feasibility and preliminary biological safety were verified in *ex vivo* pig brain and *in vivo* animal models (Wu J. et al., 2024) (Figure 6A). This model effectively improves localization accuracy and therapeutic efficacy while overcoming the structural limitations that traditional drug delivery devices face in penetrating the BBB.

As an alternative to cranial delivery, the intranasal route to the brain has gained attention. A review of studies indicated that combining the nasal pathway with hybrid nanomedicine techniques—such as magnetic navigation, low-intensity focused ultrasound, or photobiomodulation pre-treatment—could serve as a potential route for BBB-crossing drug delivery. This pathway leverages the natural anatomical channel from the nasal cavity to the brain, enabling less invasive delivery while avoiding cranial intervention (Won et al., 2023). However, its effectiveness and safety still require further validation, particularly with respect to complex anatomical structures, mucosal fluid dynamics, and delivery localization control (Torlakcik et al., 2021) (Figure 6B).

In neurosurgery, spinal cord stimulation therapy has introduced the use of magnetic-guided catheters and nanorobots to reduce electrode implantation trauma and enable remote, wireless neural modulation. However, this strategy faces critical challenges, such as generating strong magnetic fields *in vivo*, achieving precise localization, and ensuring the use of degradable, biocompatible robotic materials.

In orthopedics, particularly in the treatment of spinal metastases, massive bleeding and postoperative recurrence are major causes of surgical failure. To address these issues, a study proposed an injectable nanorobot-silk fibroin hydrogel composite (nanorobot–hydrogel superstructure). This system exhibits thixotropic properties, facilitating diffusion within tissues. The composite is injected subcutaneously into the tumor region before surgery, and after near-infrared (NIR) activation, the nanorobots self-propel into the deep tumor, releasing thrombin on demand to embolize the tumor’s blood supply. This is then combined with gold nanorods for photothermal ablation to suppress the tumor. The system works as follows: regenerative silk fibroin nanofiber hydrogels, carrying the nanorobots, are activated by NIR to actively move deep into the bone metastasis microenvironment, release thrombin to induce local microvascular embolization, and simultaneously use gold nanorods for photothermal therapy. This approach achieves the dual goals of hemostasis and tumor control, significantly reducing intraoperative blood loss, lowering postoperative recurrence rates, and improving surgical operability. It represents an innovative paradigm for “hemostasis–anti-tumor integration” in orthopedic settings (Chen et al., 2024) (Figure 6C). Despite the promise of crossing biological barriers, safety remains the paramount concern in these distinct fields. In neurosurgery, even minimal neurotoxicity from nanomaterials or their degradation byproducts can cause irreversible damage to glial cells and neurons. In orthopedics, a key challenge is ensuring that tumor-suppressive nanorobots do not impair normal osteogenesis, necessitating a precise balance between cytotoxicity against cancer cells and biocompatibility with bone marrow stem cells (Chen et al., 2024; Torlakcik et al., 2021).

## 4 Nanorobot clinical translation: biosafety, ethics, and regulatory challenges

### 4.1 Biohybrids and biosafety

Biohybrid nanorobots significantly enhance *in vivo* task execution efficiency and biocompatibility by incorporating the physical surface structures and biological functional properties of natural cells or vesicles. For instance, in the case of bacterial-derived outer membrane vesicles (OMVs), researchers have fixed urease on the OMV membrane surface, using urea to catalyze the production of ammonia and carbon dioxide, which drives the autonomous movement of the OMV-nanorobots. This system retains the natural biocompatibility, immunogenicity, and surface modifiability of OMVs, allowing for effective siRNA loading to resist nuclease degradation. Additionally, membrane-penetrating peptides on the surface enhance tumor tissue penetration. Through this design, OMV-nanorobots achieved significant *in vivo* delivery efficiency and immune-stimulating effects in a bladder orthotopic tumor model (Tang et al., 2024). However, despite demonstrating excellent biocompatibility and targeting ability, this system still faces challenges in clinical translation, including the standardization of OMV production, controlling immune system reactions, and integrating with existing treatment methods (Table 2).

**TABLE 2:** Summary of the advantages, limitations, and translational potential of the nanorobotic strategy.

Biomimetic system	Driving mechanism	Key advantages	Applications	Clinical translation challenges	References
OMV-based Urease Nanorobots	Urea-catalyzed propulsion	Strong biocompatibility, immunogenicity adjustable, high endocytosis efficiency	Tumor therapy, immune stimulation	Immunocompatibility and stability	Tang et al. (2024), Bacterial outer membrane vesicle nanorobot
Spermbots	Sperm natural propulsion	Precise control, strong fluid adaptation	Assisted reproduction, precise fertilization	Biocompatibility, effectiveness, ethical issues	Zhang et al. (2024b), Spermbots and their applications in assisted reproduction
Neutrophil Membrane-inspired Nanorobots	Neutrophil membrane disguise + copper-based nanoparticles	Antioxidant, iron clearance, anti-inflammatory, organ protection	Acute kidney ischemia-reperfusion protection	Precise localization, stability *in vivo*	Ding C. et al. (2023), Neutrophil membrane-inspired nanorobots
Platelet Membrane-covered Magnetic Nanomotors	Magnetic spiral propulsion + platelet membrane	Long-term propulsion, specific adhesion to targets	Blood therapy, toxin/bacteria isolation	Magnetic field strength, precise positioning, degradability	Li et al. (2018), Biomimetic platelet-camouflaged nanorobots
Bioresorbable Acoustic microrobots	Acoustic propulsion	Biodegradable, hydrolytic degradation reduces long-term residue	Reduce residual damage, lower long-term risks	Material selection, degradation time control	Han et al. (2024), Imaging-guided bioresorbable acoustic hydrogel microrobots

In the field of assisted reproduction, sperm-driven biohybrid robots (spermbots) have emerged as a promising technology for precise fertilization treatment, leveraging their natural propulsion abilities and fluid adaptability. Yixuan Zhang et al. thoroughly explored the possibility of combining sperm cells with nanotechnology, achieving automated precise fertilization treatments by integrating sperm cells with mechanical/nano structures. The article also proposes the “three laws” of “excellent genetics, gentle operation, and no contamination,” outlining a three-step translation route from *in vitro* validation to animal models and human clinical trials. Despite progress in in vitro research, key challenges for clinical translation include biocompatibility, controllability of treatment effects, ethical concerns, and long-term outcome validation (Zhang Y. et al., 2024).

In organ protection and inflammation suppression, membrane camouflage technology has shown broad potential for protecting organs from damage. Chenguang Ding et al. used neutrophil membrane-camouflaged copper-based nanoparticles (N-Cu_5_._4_O@DFO NPs). This system demonstrated excellent antioxidant, iron ion removal, and anti-inflammatory capabilities in an acute kidney ischemia-reperfusion (I/R) injury model (Ding C. et al., 2023). By reducing oxidative damage and inflammation, it effectively promoted kidney recovery. While this strategy shows great promise, its clinical application still faces challenges, including treatment targeting, tissue-specific enrichment, and controlling clinical drug dosages. In particular, long-term effects and personalized regulation, especially in kidney disease treatment, require more clinical data support.

Additionally, Jinxing Li et al. designed platelet membrane-coated magnetic helical nanomotors, which achieved long-term propulsion in the bloodstream and specifically adhered to toxins or bacteria, providing a “traveling isolation” function (Li et al., 2018). This design effectively reduces systemic side effects and offers more precise treatment options. However, issues such as *in vivo* stability, reusability, and cross-species biocompatibility still need to be addressed, particularly in terms of immune response control and targeting stability during prolonged propulsion.

In addition to these functional advantages, biohybrid nanorobots also show significant benefits in metabolic clearance and *in vivo* residue reduction. For example, research on absorbable acoustic microrobots has shown that using a hydrophilic/hydrophobic bilayer structure and bubble cavity design enables stable propulsion during treatment, followed by hydrolytic degradation, significantly reducing the risk of long-term residuals (Han et al., 2024). However, despite the use of degradable materials to reduce postoperative residue, challenges remain in optimizing degradation speed, ensuring stability during treatment, and achieving harmless post-treatment degradation.

From a translational perspective, several key issues must be addressed before nanorobots can enter clinical applications: immune compatibility, visualization capabilities, degradable metabolism, dosage control, and clearance mechanisms (Soto et al., 2020; Agrahari et al., 2020). These factors represent material and biosafety thresholds that need to be met. In immune-stimulation systems based on nucleic acid platforms, issues such as off-target risks, aptamer interactions, and macromolecule pharmacokinetics *in vivo* must be systematically resolved and standardized (Asumadu et al., 2025; Keller and Linko, 2020). While the biohybrid strategy offers powerful functionality and targeting capabilities for nanorobot technology, overcoming these technical and biological obstacles remains crucial for its successful application in surgical medicine.

### 4.2 Translation, regulation, and ethics

For micro/nanorobot technology to successfully transition from the laboratory to clinical practice, it must undergo a rigorous translation process that includes meeting various requirements, from technical performance verification to compliance with ethical and regulatory standards. Several reviews have emphasized that for nanorobots to be widely adopted in clinical applications, they must meet a series of key standards: biocompatibility, visualization capability, biodegradable materials, controllability of dose-effect-exposure, and shutdown/clearance mechanisms (Soto et al., 2020; Sun et al., 2024; Agrahari et al., 2020; Rui et al., 2025). These standards provide the foundation for ensuring the safety and efficacy of nanorobots in clinical settings, allowing them to perform their intended tasks effectively and stably while minimizing the potential for long-term residual risks to patient health. Additionally, a comprehensive regulatory and ethical framework must be developed to address the challenges that may arise during the clinical translation process (Soto et al., 2020; Agrahari et al., 2020).

From a therapeutic outcomes perspective, a complete efficacy-safety chain, extending from animal studies to human clinical trials, is essential. According to various sources, early animal studies must evaluate the distribution, targeting, degradability, and biocompatibility of nanorobots in target tissues. Following this, clinical trials should include measurable clinical endpoints, such as reperfusion time, margin error rates, tumor suppression rates, intraocular pressure changes, and organ function protection. These clinical indicators not only reflect treatment efficacy but also provide insights into the short- and long-term effects of nanorobots on patients.

However, the translation of micro/nanorobot technology also faces significant ethical challenges and privacy protection issues. For example, in the case of intraoperative navigation platforms based on SERS technology, the use of SERS provides real-time molecular decision-making support during surgery, but it also involves the real-time collection and processing of patient biological data (Chen et al., 2025). As a result, privacy protection and data governance become critical concerns. This necessitates that the intraoperative navigation system must be transparent and traceable to ensure patient privacy and data security, while also safeguarding the decision-making responsibility of healthcare professionals. In applications within the reproductive system and central nervous system, ethical considerations, multi-center collaboration, and long-term follow-up monitoring are particularly important. The use of nanorobots in these fields, especially in assisted reproduction and central nervous system disease treatment, requires heightened attention to their long-term impact on the human body and the representativeness of the patient population. For example, when sperm-driven nanorobots (spermbots) are used in assisted reproduction, one of the primary clinical translation challenges is addressing the ethical concerns, alongside ensuring biocompatibility and treatment efficacy (Zhang Y. et al., 2024).

Additionally, safety and regulatory risks associated with different propulsion and carrier platforms must be thoroughly addressed. For instance, the *in vivo* localization accuracy of chemical propulsion systems, fuel safety, immunogenicity and off-target effects of nucleic acid platforms, and the immune responses and long-term host effects associated with membrane camouflage systems must all undergo rigorous toxicological studies and pharmacokinetic assessments (Asumadu et al., 2025; Ding C. et al., 2023; Peng et al., 2023). Such research will provide the necessary scientific evidence to support the widespread clinical use of nanorobots, ensuring that side effects and toxic reactions are effectively mitigated, thereby safeguarding patient safety.

Table 2 provides a systematic comparison of the actuation mechanisms, advantages, and target applications characterizing various biomimetic nanorobotic strategies. This analysis elucidates a core characteristic of the field alongside two primary translational challenges. First, a distinct translational paradigm emerges from this technological diversity: enhancements in biocompatibility and targeting efficacy—exemplified by cell membrane camouflage and biohybrid systems—are frequently achieved at the expense of increased manufacturing complexity and uncertainty regarding biological behavior. For instance, while neutrophil- or platelet-based platforms offer inherent targeting advantages, their standardized fabrication and long-term *in vivo* stability remain persistent challenges. Second, this technical trade-off precipitates a marked disparity between theoretical potential and current clinical status. As detailed in Table 2, while relatively straightforward targeted delivery applications have advanced to late preclinical or early clinical trials, systems designed for complex active interventions (e.g., microsuturing, ablation) remain largely at the *in vitro* proof-of-concept stage. This suggests that the primary clinical bottleneck has evolved from merely demonstrating basic “locomotion capabilities” to the more intricate challenge of holistic system integration. The crux of this shift lies in resolving issues related to real-time imaging feedback compatibility and the predictability of long-term *in vivo* behavior, such as biodegradation kinetics. Consequently, advancing these frontier strategies toward clinical application necessitates not only continuous technical optimization to reconcile intrinsic trade-offs but also urgent, parallel breakthroughs in standardized verification protocols and scalable, safety-controlled manufacturing processes.

In summary, the transition of nanorobots from laboratory research to clinical practice involves not only technological advancements but also comprehensive considerations of regulations, ethics, and safety governance. These challenges must be addressed concurrently during the translation process to ensure that nanorobots can maximize their effectiveness in real-world clinical applications and provide tangible therapeutic benefits to patients. In the future, with the continued improvement of standardized processes and ethical frameworks, nanorobots are expected to become reliable auxiliary tools in various medical fields, particularly in surgical medicine.

## 5 Future prospects

**FIGURE 7:**
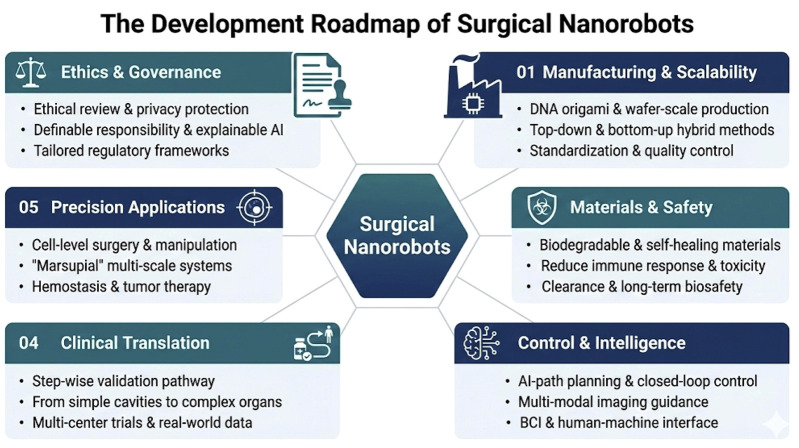
Future challenges and prospects in surgical nanorobotics.

As a transformative and cutting-edge technology, nanorobots offer unprecedented opportunities for precise treatment and minimally invasive interventions in surgical medicine. However, despite significant advances in manufacturing processes, material innovations, and the integration of multimodal technologies in recent years, several key challenges remain (Figure 7).

### 5.1 Potential applications of nanorobots in surgical medicine

The successful transition of nanorobot technology into clinical applications depends not only on technological breakthroughs but also on manufacturing capabilities, system consistency, and scalability. While DNA nanoplatforms offer high programmability and molecular precision, challenges such as high costs, low throughput, and complex purification processes persist in large-scale manufacturing. To address this bottleneck, some studies have proposed combining enzymatic tools with life-like artificial systems to improve large-scale preparation efficiency and reproducibility of DNA structures (Abdollahzadeh et al., 2025). Additionally, nanorobots driven by acoustic bubble mechanisms have already achieved production at the wafer scale, with over 10^9 units and precise control of sizes ranging from 500 nm to several microns. This provides important technological demonstrations for process reproducibility and industrialization (McNeill et al., 2020).

In terms of manufacturing methods, the production of magnetic nanorobots involves both “top-down” and “bottom-up” approaches, creating a diversified manufacturing spectrum. Top-down methods include macro manufacturing technologies such as injection molding, material self-rolling, melt electrostatic writing, deposition, and photolithography, while bottom-up methods, such as chemical synthesis, self-assembly, and 3D printing, offer additional choices at the micro/nanoscale. This dual-track manufacturing strategy provides multiple possibilities for scaling, cost control, and functional integration of nanorobots in the future (McNeill et al., 2020). The establishment of standardized reporting, performance evaluation systems, commercialization pathways, and risk control mechanisms is essential for transitioning from the laboratory to the market (Zhang et al., 2025). These measures can effectively reduce investment and translation risks during commercialization (Soto and Chrostowski, 2018). Furthermore, microfluidic technology plays a critical role as the “production line” and “test bench” for micro-actuators and microsystem integration, enabling continuous material gradient generation, structural refinement, and multi-signal stimulation (Ma et al., 2023).

### 5.2 Key technological breakthroughs and challenges

In materials science, the concurrent advancement of absorbable and self-healing materials significantly enhances the stability and long-term serviceability of nanorobots *in vivo*. For example, absorbable acoustic microrobots with hydrophilic/hydrophobic bilayer structures and bubble cavity designs can achieve stable propulsion during treatment and later undergo hydrolytic degradation, greatly reducing the risk of *in vivo* residue (Han et al., 2024). Self-healing materials, meanwhile, show great potential in autonomous robots and wearable electronic devices. These materials enhance damage resistance, repair, and long-term serviceability of micro/nanosystems in complex *in vivo* environments through multi-scale reversible bonding and integrated conductivity/structure (Tan et al., 2021).

Regarding control systems, future nanorobots may evolve from the current imaging-AI-surgeon interface toward a neurocontrol and brain-machine interface (BCI) closed-loop system. However, this process must carefully navigate ethical concerns and the risks associated with invasive interfaces, integrating imaging, AI, and the surgeon interface to ensure safety and ethical compliance in clinical practice (Rui et al., 2025; Grezenko et al., 2023). In industrial nanorobots, Pfeiffer et al. made a breakthrough by incorporating DNA origami structures as energy-supplying hardware modules, offering new opportunities for device-internal manufacturing (Soto et al., 2020). Meanwhile, Zhou et al. demonstrated the application of 3D DNA industrial nanorobots for light/temperature dual-control, multi-axis positioning, grabbing, welding, and self-replication, marking the transformation of nanorobots into autonomous manufacturing platforms (Siti et al., 2023; Liu et al., 2023; Zhou et al., 2023).

### 5.3 System engineering and standardization

To meet the practical needs of intraoperative applications, a three-step translation roadmap can be outlined. The first step, the “visualizable—controllable—disappearable” verification stage, focuses on treatment scenarios with good imaging and accessibility, such as the abdominal cavity, luminal structures, and the eye. This stage aims to verify the safety and preliminary efficacy of micro/nanorobot systems. The second step is the “cross-scale coordination” system integration stage, which includes key technical paths like collaboration between “mother” and “offspring” robots and multi-modal imaging with AI closed-loop control, addressing high-difficulty scenarios such as the brain, spinal cord, and blood vessels (Wu J. et al., 2024; Vartholomeos et al., 2011). The third step, the “standard—production line—ethics” concurrent clinical expansion stage, involves establishing manufacturing standards, quality assessments, intraoperative workflow protocols, and ethical governance frameworks. This will drive the expansion of indications from single-center to multi-center trials and facilitate real-world evaluations (Soto and Chrostowski, 2018; Rui et al., 2025).

This translation process requires the synchronized advancement of all stages, especially in regulation, ethics, and safety, ensuring that nanorobots have measurable and controllable safety and efficacy standards throughout their journey from laboratory research, industrial manufacturing, animal models, to human clinical trials. Only through comprehensive governance of manufacturing, materials, control, ethics, and regulations can nanorobots become reliable tools in surgical medicine, promoting the development of precision and personalized medicine.

### 5.4 Accumulation of clinical translation and animal experiment data

In cell manipulation and targeted biopsy, untethered microgrippers, as a key component of the nanorobot system, have demonstrated high adaptability in complex cavities. These devices can perform tasks such as grabbing, twisting, and cutting, offering new solutions for tissues that are difficult to access during surgery. This development marks a significant advancement, indicating that nanorobots can provide precise operations in minimally invasive surgery and play a crucial role in tasks at the cellular and subcellular levels (Zhou et al., 2024). Further research, such as that by Jiang Yan et al., demonstrated the use of vector Lorentz force-driven single nanowire deformation grippers, capable of performing multiple operations like grabbing, twisting, and high-frequency vibration. This overcomes van der Waals adhesion forces at the microscale, offering a new paradigm for high-precision assembly and biological manipulation (Yan et al., 2023).

With advancements in intelligent materials and control systems, future nanorobots will play a key role in more complex tasks, particularly in precision medicine and biomolecular regulation. The enhancement of nanorobot precision and functionality will offer new solutions for biomolecular assembly, drug delivery, and disease treatment.

### 5.5 Integration of multimodal imaging and closed-loop control

At the cellular and subcellular levels in surgical medicine, nanorobot technology has shown immense potential in precise cellular manipulation and subcellular structural remodeling. Atomic force microscopy (AFM)-based nanorobotic surgery platforms have already enabled the cutting and reshaping of intermediate filaments inside living cells. This technology facilitates the “cell-mechanics-connection” pathology simulation, offering new perspectives for the precise manipulation of intracellular structures (Yang et al., 2015). This breakthrough enables nanorobots to perform extremely fine operations at the cellular level, advancing the study of cellular mechanics and behaviors, particularly in tumor cell migration, cell-cell connection interference, and cell reprogramming.

Simultaneously, optical tweezers and nanorobot-assisted integrated systems provide an efficient platform for single-cell operations. In the “video feedback-force/position closed-loop” control mode, nanorobots can use optical tweezers to cut, analyze, and manipulate single cells with high precision (Shang et al., 2016; Xie et al., 2018). This system integrates real-time video feedback with mechanical control, allowing the operator to perform high-precision operations at the microscopic scale while dynamically adjusting the applied force and displacement, ensuring both surgical precision and safety. As optical tweezers technology and robotic systems continue to integrate, future applications will expand to cell biology, molecular biology, and personalized treatments, especially in single-cell analysis, gene editing, and cell repair.

In nanorobot development, future research will continue to optimize dual-arm nanorobot systems, which have already made breakthroughs in minimally invasive surgery. By synchronizing rotation suture trajectory design, these systems significantly reduce suture deformation and slippage, improving mechanical strength and reliability (Jiang et al., 2025). As technology progresses, future dual-arm nanorobots will have greater degrees of freedom and precision, capable of tackling more complex and diverse surgical tasks, especially in multi-tissue micro-incision procedures and multimodal imaging environments.

### 5.6 Intelligent path planning and dynamic adaptability

At both the macro and micro levels, the integration of AI path planning and visual closed-loop control systems enables nanorobots to operate in a “perception-decision-execution” three-link system, greatly enhancing their autonomy and path optimization capabilities. This technological advancement will allow nanorobots to autonomously navigate and avoid obstacles in dynamic, complex environments, providing strong support for precise clinical surgeries. For example, research by Chang Yuchou et al. demonstrated the application of the A* path planning algorithm in vascular networks under magnetic resonance angiography (MRA) guidance, offering technical support for high-difficulty surgeries, such as those in the brain and vascular systems (Chang et al., 2018).

As technology advances, nanorobots will not only perform precise, fine operations but also possess the ability to re-plan and make real-time decisions in dynamic environments. This will significantly enhance their reliability and safety in complex surgeries. However, with the rapid development of nanorobot technology, future research will face challenges, particularly in high-risk surgical environments. Ensuring the long-term safety and stability of these systems during complex, high-risk procedures is a critical technical challenge. Moreover, ensuring accountability during real-time decision-making and achieving the explainability of AI algorithms will be essential for the clinical application of nanorobots (Grezenko et al., 2023).

As nanorobot technology progresses, the expansion of its application will inevitably raise sensitive issues such as ethics and privacy protection. Therefore, regulating the ethical review and supervision of AI and nanorobots in clinical applications will be crucial for ensuring their safe and legal use, ultimately promoting their widespread adoption.

### 5.7 Improving ethical, privacy, and regulatory frameworks

Advancements in materials are also a key driver of nanorobot development. Future nanorobots will rely heavily on innovations in absorbable and self-healing materials. For instance, the absorbable acoustic microrobots system, with its hydrophilic/hydrophobic bilayer structure and bubble cavity design, provides stable propulsion during treatment and reduces *in vivo* residue risk through hydrolytic degradation post-treatment (Han et al., 2024). Additionally, with the progress of self-healing materials, nanorobots will be able to self-repair in extreme environments, restore functionality, and enhance long-term serviceability within the body (Tan et al., 2021). These material innovations will greatly advance the application of nanorobots in complex biological environments, particularly in remote monitoring and long-term treatments, further expanding their clinical application scenarios.

Surgical robot technology has already matured in fields such as orthognathic surgery, abdominal surgery, and urology. Looking ahead, the integration of nanorobots with these mature surgical robots will transform the “microsurgical incision—molecular-level operation” workflow. By deeply integrating nanorobots with AI, AR, and multimodal imaging technologies, future surgical systems will further enhance treatment precision and safety (Chatterjee et al., 2024; Grezenko et al., 2023; Lin et al., 2023). In minimally invasive scenarios such as root canal treatments and orthodontics, nanorobots show enormous application potential. When combined with AI diagnostics and haptic feedback systems, nanorobots are expected to play a key role in minimally invasive disinfection and remote monitoring (Adil et al., 2025; Adel et al., 2021).

In summary, the integration of nanorobots with AI is set to revolutionize the medical field, particularly in minimally invasive surgery, precision medicine, and cell therapy. As technology, materials, and control systems continue to progress, nanorobots will become essential auxiliary tools in surgical medicine, improving surgical precision, reducing patient trauma, and accelerating treatment processes. However, to achieve this, challenges related to technology, ethics, law, and safety must be addressed to ensure these innovative technologies can be safely and effectively applied in clinical practice, ultimately improving patient health outcomes.

## 6 Conclusion

In conclusion, nanorobotic technology exhibits transformative potential in surgical medicine, particularly within the domains of minimally invasive surgery and precision therapy. However, the transition from laboratory prototypes to routine clinical practice necessitates overcoming a series of complex interdisciplinary barriers. Current bottlenecks are primarily centered on technical integration and system verification.

First, regarding manufacturing and materials, while self-healing matrices and biohybrid strategies offer promising solutions for stability and longevity, significant challenges remain. The scalable, cost-effective fabrication of complex platforms—such as DNA nanorobots—persists as a core obstacle to mass production. Furthermore, the long-term *in vivo* degradation kinetics of these novel materials and the potential toxicity of their byproducts require clarification through rigorous longitudinal safety assessments to mitigate the risk of unpredictable immunogenic responses.

Second, in the realm of control and imaging, the deep integration of artificial intelligence with multimodal imaging is poised to significantly enhance surgical precision. Nevertheless, achieving precise, real-time actuation of magnetic and acoustic systems within dynamic physiological environments (e.g., high-velocity blood flow) is currently impeded by a fundamental trade-off. Specifically, limitations in deep-tissue imaging resolution, feedback signal latency, and the computational latency associated with processing volumetric 3D data collectively hinder the realization of reliable closed-loop control.

Finally, at the level of clinical translation, non-technical barriers remain prominent despite a clear roadmap toward personalized medicine. Beyond technical hurdles, the absence of standardized regulatory frameworks and comprehensive efficacy evaluation systems constitutes a significant impediment to clinical adoption. Concurrently, governance issues arising from increased system autonomy, including data privacy, algorithmic ethics, and long-term biosafety, must be systematically addressed within established legal and ethical frameworks.

Overall, future breakthroughs will depend on synergistic innovation: on one hand, overcoming technical bottlenecks related to stability and adaptive intelligence through advanced manufacturing and AI-driven multimodal image fusion; and on the other, establishing a comprehensive evaluation ecosystem that encompasses technical efficacy, biosafety, and ethical standards. While nanorobots are poised to play a transformative role in the future of surgery, the key to genuine clinical translation lies in shifting the research focus from isolated proof-of-concept demonstrations to addressing the fundamental challenges of system integration and clinical validation. This shift is essential to steadily propel surgical medicine toward a new era of precision and personalization.
