# Supplementary material for: Advancements and challenges of nanorobots in surgical medicine: design, applications, and interdisciplinary integration
Source: Front Bioeng Biotechnol. 2026 Mar 24;14:1778916. doi: 10.3389/fbioe.2026.1778916 (PMC13055592; doi:10.3389/fbioe.2026.1778916)
Supplement: Supplementary file 1 [file Supplementaryfile1.docx]

Supplementary Material

This document aims to provide a transparent and detailed explanation of the use of generative artificial intelligence tools to assist in manuscript preparation for the article Nanorobotics Advances and Challenges in surgical Medicine: design, application, and interdisciplinary integration. All initial content generated by the AI tool, which was used only as an aid, underwent rigorous, lead human review, editing, fact-checking, and scientific validation by the authors. The authors assume full responsibility for all content, scientific accuracy, completeness, and academic views of the final manuscript.

**1. The use of AI in illustration generation**

**1.1 Tools and core workflow**

Our illustration generation is an author-led, multi-step, iterative workflow, with AI providing assistance at the initial stage of concept visualization. First, the author used DeepSeek AI to analyze the content and logic of specific chapters of the manuscript, aiming to transform the complex scientific description into structured English prompt words that can be used to guide image generation. This process involves multiple interactions between the author and the AI, and the author controls the final composition logic through instructions.

The following is the corresponding chat history of Deepseek:

Question from the author: Hello, you are now an experienced scientific research graphic designer, I am going to draw illustrations for my review, this is the content of my review, please output an English prompt for my review according to the content to facilitate my drawing, I am going to draw a large picture for this part of the content, but how many small pictures need to be decided by you. Figure 1A, Figure 1B and so on. Please output the corresponding prompt to me in turn.

Deepseek's answer:Based on the content of your review on tumor surgery and local therapy, I have designed a comprehensive figure that illustrates the key applications of nanotechnology and nanorobots in this field. The figure is divided into 7 subfigures (Figure 1A to 1G), each highlighting a specific technology or strategy described in your text. Each subfigure prompt is designed to be used for generating a scientific illustration or schematic diagram, focusing on clarity, mechanism depiction, and visual impact. Below, I provide an overall description for the big figure, followed by individual prompts for each subfigure.

Overall Big Figure Description:

Title: "Advanced Nanorobotic Platforms in Tumor Surgery and Local Therapy"
Description: This figure summarizes the integration of smart nanorobots and nanotechnology in enhancing tumor surgical outcomes and local therapies. It includes multiple subfigures demonstrating various applications such as intraoperative margin identification, peritoneal metastasis treatment, pre-operative hemostasis, chemodynamic therapy, immune activation, targeted drug release, and ocular tumor management. The illustrations should use a consistent style (e.g., schematic diagrams with labels, arrows for processes, and color-coding for different components) to show the mechanisms, workflows, and key benefits like reduced decision time, minimized off-target effects, and improved efficacy.

Individual Prompts for Subfigures:

Figure 1A: SERS-Active Smart Nanoplatform for Intraoperative Margin Identification
Prompt: "Create a scientific illustration showing a tumor surgical site with uncertain margins. Depict SERS-active smart nanoprobes (e.g., nanoparticle clusters) accumulating in the tumor microenvironment, emitting amplified signals for real-time warning. Include an augmented reality (AR) navigation system overlay displaying molecular-morphological data, forming a closed-loop workflow. Show arrows indicating signal amplification and reduced decision delay. Highlight the probe self-navigation and enrichment in the tumor area, with labels for key elements like SERS probes, AR interface, and tumor tissue."

Figure 1B: Magnetic-Laser Integrated System for Peritoneal Metastasis
Prompt: "Illustrate a peritoneal metastasis model in a abdominal cavity. Show magnetic nanorobots (e.g., DOX-loaded nanoparticles) being driven by an external magnetic field to aggregate against gravity. Include an ultrasound probe for guidance and a near-infrared (NIR) laser triggering localized photothermal amplification and drug release. Depict multiple tumor foci receiving on-demand treatment, with heat waves representing photothermal effects and reduced off-target thermal damage. Label components such as magnetic nanorobots, NIR laser, ultrasound, and tumor nodules."

Figure 1C: Injectable Nanorobot-Silk Hydrogel Composite for Pre-operative Hemostasis
Prompt: "Draw a schematic of a highly vascularized tumor (e.g., in a mouse vertebral model) before surgery. Show an injection of nanorobot-silk hydrogel composite containing thrombin and gold nanorods. Depict NIR light penetrating deep into the tumor, inducing thrombin release for microvascular embolism and photothermal inhibition. Use arrows to represent NIR-triggered penetration and coagulation, with labels for thrombin, gold nanorods, hydrogel, and reduced bleeding. Highlight the minimal invasive pre-operative effect and decreased postoperative recurrence."

Figure 1D: Multi-enzyme Cascade Magnetic Nanorobots for Chemodynamic Therapy
Prompt: "Create a diagram of a solid tumor interior with multi-enzyme cascade magnetic nanorobots. Show glucose oxidase (GOx) producing hydrogen peroxide (H2O2), which then reacts with peroxidase-like activity to generate hydroxyl radicals (•OH). Include copper ions depleting glutathione (GSH). Depict an external magnetic field enhancing targeted accumulation in the tumor. Use color changes or bubbles to represent reactive oxygen species and GSH depletion, with labels for GOx, •OH, Cu ions, and magnetic field. Emphasize the improved chemodynamic efficacy."

Figure 1E: Magnetic Nanorobots with Immune Activation for Breast Tumor
Prompt: "Illustrate a breast tumor treated with magnetic nanorobots that induce immunogenic cell death through chemical catalytic reactions. Show the nanorobots releasing agents that damage tumor cells, leading to the release of antigens and activation of dendritic cells and T-cells. Depict a systemic anti-tumor immune response with arrows indicating the cascade. Include a graph or icon showing a 59.3% inhibition rate. Label key elements like magnetic nanorobots, immune cells, and cytokine release."

Figure 1F: DNA Nanorobots for Smart Recognition and On-demand Release
Prompt: "Draw a schematic of DNA origami and nucleic acid computing platforms for targeted oligonucleotide drug release. Show DNA nanorobots recognizing tumor-specific markers and releasing drugs on demand. Include elements like enzymatic amplification and simplified processes for scalable production. Depict integration with machine learning for personalized precision therapy, using circuit-like diagrams for nucleic acid logic. Label components such as DNA折纸, drug payload, and ML algorithms."

Figure 1G: Intravitreal Injection and Nanorobots for Ocular Tumor Therapy
Prompt: "Create an illustration of the eye with ocular tumor and neovascularization. Show intravitreal injection of smart responsive nanorobots into the vitreous humor. Depict the nanorobots actively regulating neuro-vascular interactions, with arrows indicating targeted delivery and response to stimuli. Highlight this as a next-generation direction for intraoperative and perioperative treatment. Label key parts like vitreous body, tumor,New blood vessels, and nanorobots."

These prompts are designed to be detailed yet flexible for generating high-quality illustrations. You can adjust them based on specific software or style preferences. If you need modifications or additional subfigures, let me know!

Question from the author: Now I am going to draw the mechanism diagram of the review, please generate two corresponding abstracts according to the content of my review. This part is going to draw two figures, respectively, Figure 1A and Figure 1B: (manuscript text)

Deepseek's answer: Based on the review content you provided, I have generated two summaries of the mechanism diagrams for you, corresponding to Figure 1A (nanorobot lineage and design tradeoff) and Figure 1B (intraoperative application scenario and system integration).

Figure 1A Abstract: Technology spectrum and core design tradeoffs of surgical nanorobots

This figure illustrates the four technology lineages of surgical nanorobots and the core elements that need to be balanced during design.

Technology spectrum:

Artificial nano-robots: represented by magnetic chain, acoustic and optoelectromagnetic coupling robots, emphasize propulsion stability, programmable control and image visualization [11][20][10].

DNA nanorobots: Use origami structure and nucleic acid logic to realize molecular level environment sensing, Boolean operation and on-demand release, with high programmability [1][3][5][69].

Biological hybrid: By coupling life components such as outer membrane vesicles, sperm and bacteria, robots are endowed with intelligence such as chemotaxis, autonomous movement, immune regulation and camouflage [24][25][118][45].

Swarm and reconfigurable microswarm: through the interaction of external field coding, cluster morphology switching, cooperative division of labor and closed-loop control are realized [29][82][97].

Core design trade-offs:

The development of the four technical lineages needs to be balanced around the following four core dimensions under strict intraoperative constraints:

Function (e.g., on-demand release vs. metabolic clearance)

Materials (e.g., drive efficiency vs. biodegradability)

Imaging (e.g., multimodal visualization vs. material compatibility)

Control (e.g., active drive accuracy vs. in vivo energy coupling efficiency)

This figure aims to reveal that from a single component to an intelligent cluster, its design is a system engineering that pursues performance optimization under multiple constraints [20][90][31].

Figure 1B Abstract: Intraoperative application scenario of surgical nanorobots and system synergy

This figure demonstrates the task execution of the nanorobot in four high-value intraoperative scenarios and its collaborative workflow with the macroscopic surgical system.

Intraoperative application scenarios:

Identification and navigation: Through SERS, photoacoustic, ultrasound, MRI and other multimodal imaging, the molecular boundary of lesions can be calibration and localization, and integrated with augmented reality navigation system to provide visual guidance for surgeons [6][79][34][145].

Hemostasis and microenvironment remodeling: local precise regulation of acute hemorrhage and ischemia-reperfusion injury can be achieved by targeted delivery of thrombin or implementation of antioxidant intervention [22][45].

Local treatment and combination therapy: the implementation of photothermo-chemotherapy synergy, enzyme dynamic therapy, etc., to achieve spatiotemporal coupling of on-demand treatment and immune synergy [36][18][42].

Miniaturized operation: as a nanosurgery toolbox, it performs fine operations such as stitching, grasping and cutting at the tissue and cell level [14][40][133][136].

System integration and collaboration:

The successful implementation of all application scenarios depends on the effective collaboration of the nanorobot platform with the macroscopic surgical system, artificial intelligence planning algorithms, and the operator's operator interface, forming a complete clinical workflow from image guidance to closed-loop control, and finally realizing real-time visualization to meet the stringent intraoperative requirements.

Author's question: Not a summary of the mechanism diagram, but a summary of the illustrations

Deepseek's answer: OK, I totally understand. This time is a summary of the review illustrations themselves, used to guide the drawing and to illustrate in the figure notes. The following is a summary of the illustrations written for Figures 1A and 1B, respectively, focusing more on the composition and presentation logic of the visual elements.

Illustration Summary: Figure 1A | Technology spectrum and design tradeoff of surgical nanorobots

Visual concept: A four-quadrant or layered central radiogram is used to showcase the technology spectrum of the four nanorobots, culminating in a central design trade-off area.

Composition elements and layout suggestions:

Four technical branches (in the four corners or around) :

Artificial nanorobot area: Drawing representative robot morphologies such as magnetic nanochains, microbubbles driven by sound waves, and swarms of particles subjected to light/electromagnetic fields. "Programmable control" is represented by a dashed arrow, and "image visualization" is represented by an MRI or ultrasound icon.

DNA nanorobotic Zone: Drawing a DNA origami structure as a carrier that contains molecular switches that sense environmental signals such as pH, enzymes on its surface or inside. Boolean operations are represented by logical gate symbols (AND, OR) and the dynamic process of "on-demand release" of drug molecules from the structure is depicted.

Biohybrid zone: Plots a mixture of magnetic particles bound to bacterial flagella, or a drug-loaded nanoparticle encapsulated by the cell membrane. Chemical gradient lines were used to represent "chemotaxis", and immune cells were added to the background to reflect "immune regulation and camouflage".

Swarm and Reconfigurable Microswarm area: depicting a group of identical nanounits that self-assemble from a dispersed state into a specific preprogrammed structure (such as a network or rod structure) under the guidance of a magnetic or light field to demonstrate "morphological switching" and "cooperative division of labor".

Central intersection area (design tradeoff) :

Point the arrows of the above four regions to a central region.

In the center, the core design tradeoffs are highlighted with four interrelated ICONS or keywords: "function" (e.g., a gear), "material" (e.g., a molecular structure), "image" (e.g., an eye icon), and "control" (e.g., a gamepad).

The four elements are connected by a two-way arrow, indicating the need for balance and compromise between them.

Purpose of Illustration: This figure aims to visually present the representative morphology and capabilities of the four major nanorobotic technology routes, and to illustrate the core design challenges and trade-offs they all face.

Illustration Summary: Figure 1B | Intraoperative application of surgical nanorobots with system integration

Visual concept: A circular workflow diagram was used with the surgical area in the center and the outer ring demonstrating four consecutive clinical application scenarios and emphasizing integration with the macroscopic system.

Composition elements and layout suggestions:

Central operating area:

Draw a simplified profile of human tissue containing targets that require intervention, such as tumors, blood clots, and bleeding points.

Outer ring application scenarios (in clockwise order) :

Scenario 1: Identification and navigation

Depicting nanobots, such as SERS tags, congregating at tumor boundaries and emitting specific signals.

These signals are captured by external multimodal imaging devices such as MRI, ultrasound probes.

Imaging data flow into a screen of augmented reality glasses that superimpose the molecular boundaries of the tumor, enabling visual navigation.

Scenario 2: Hemostasis and microenvironment remodeling

Nanobots are depicted targeting a vascular lesion and releasing thrombin to form a fibrin mesh for hemostasis.

Or in another area, the robot releases antioxidants to neutralize a flashing symbol representing reactive oxygen species, indicating intervention against ischemia-reperfusion injury.

Scenario 3: Local treatment versus combination therapy

The nanorobots, activated by an external field (such as near-infrared light), are depicted to simultaneously produce photothermal effects (represented by heat waves), release chemotherapeutic drugs (small molecule symbols), and induce immunogenic cell death (represented by dead tumor cells and activated T cells) at the tumor site.

Scenario 4: Miniaturization operation

Presented in the form of a "toolbox", it is shown that nanoscale tweezers are grasping a cell, a nanoknife is performing a cut, and a ring structure is completing a sew junction task.

System integration framework:

In the background or top layer of the whole ring, the macroscopic surgical system, the AI planning algorithm, and the operator's operation interface are represented by a block diagram. They are connected with the whole intraoperative application loop through data flow and control flow to form a closed-loop control system.

Illustrationpurpose: This figure aims to dynamically demonstrate how a nanorobot can perform specific tasks in four intraoperative scenarios and illustrate how it can be integrated into image-oriented clinical workflows to achieve synergy with macroscopic systems and artificial intelligence.

The author's question: Very good, continue, this part of the content you need to answer first generate several abstracts, draw several figures, how to subdivide, this part of the content of the illustration is Figure 2, and then write the corresponding illustration summary as before: (manuscript text)

Deepseek's answer: OK, let's plan an illustration for this section on "Programmable intelligent platforms".

According to the text you provided, the central thread is to explain how to give intelligence to nanobots through various programmable technologies (nucleic acids, enzymes, catalytic materials). Therefore, I propose to subdivide Figure 2 into three subgraphs, corresponding to three progressive technical levels:

Figure 2A: DNA Computing and origami Framework (static programming basics)

Figure 2B: Nucleic acid track and motion device (dynamic execution and response)

Figure 2C: Enzyme cascade and catalytic Nanosatorios (chemically driven and synergistic therapy)

This division can clearly show the complete technical chain from "molecular logic design" to "motion execution" to "chemical energy drive and synergy".

The following is a summary of the corresponding illustrations for the three subfigures:

Illustration summary: Figure 2A | Programmable computing framework for DNA nanorobots

Visual concept: In the form of a modular flowchart, it shows the process of DNA computation and control from "input" to "logical processing" and then to "output".

Composition elements and layout suggestions:

Left - Input module:

Multiple molecular input signals are drawn, such as pH ICONS, enzyme molecules (scissor shapes), small molecules (aptamer targets), and light.

These signal arrows point to the central logical processing module.

Core - Logical processing core:

A large DNA origami structure, such as a rectangular or hexagonal platform, is used as a carrier.

On the origami structure, Boolean logic gates are clearly drawn, including gate symbols such as AND, OR, and NOT, and these logics are visualized with connections of DNA strands.

In particular, the CRISPR-Cas system, such as the illustration of Cas protein shearing DNA, is shown as a high-level logic module integrated into the origami platform.

"Polymorphic switches" and "sequences of time-controlled operations" are schematized by conformational changes, such as changing from a closed to an open state." Right - Output module:

From the altered DNA origami structure, functional loads such as oligonucleotide drugs, mirnas such as miR-1-3p, or anticancer small molecules are released. "Targeted release" and "signal amplification" were marked at the output.

Illustrationpurpose: This figure is designed to visually demonstrate how DNA nanorobots can be used as programmable molecular computers to sense complex biological signals and perform precise decision making and release functions by integrating multiple logic gates.

Illustration summary: Figure 2B | Nucleic acid orbital motion device with intelligent response

Visual concept: In the form of a dynamic path diagram, the motion of a DNA robot on a nanotrack and its intelligent response in a specific disease environment are shown.

Composition elements and layout suggestions:

Upper part - Motion device:

Draw a long rod-shaped DNA origami track.

Scene I: A DNA bipedal walker, illuminated by a beam of light, performs a reversible, step-by-step walk on a track, indicated by a footprint symbol and a back-and-forth arrow.

Scenario II: A DNA molecular motor performs high-speed, directional translation on a circular or straight-line origami track and can demonstrate the ability to turn at acute angles.

Second half - Environmental Response and Application:

Thrombus application scenario: Draw a vascular background filled with a high concentration of thrombin. A DNA nanorobot structure, upon sensing the thrombin threshold, undergoes a conformational change to release the anticoagulant.

Periodontitis application scenario: Draw an inflammatory tissue background with pathological fibroblasts. A DNA nanorobot targets and attaches to the cell surface and subsequently releases miR-1-3p to repress the expression of the disease-causing gene.

Illustrator Purpose: This figure aims to combine the "motion" capability of nucleic acid nanodevices with the "environmentally responsive" release function to demonstrate their application potential in dynamically performing complex tasks, such as nanofabronting, disease therapy.

Illustration Summary: Figure 2C | Catalytically driven nanorobots in synergy with reaction-motion-therapy

Visual concept: A serial diagram of "fuel consumption-motion-reaction generation" was used to demonstrate how non-nucleic acid based nanororobots can achieve autonomous driving and collaborative treatment through catalytic cascades.

Composition elements and layout suggestions:

Left-sided magnetic-enzyme cascade nanorobot (taking MPC as an example) :

A chain-like magnetic nanorobot was drawn, and the surface was equipped with the active sites of glucose oxidase and peroxidase-like enzymes.

Directional motion under magnetic field guidance is indicated by arrows.

Demonstrating its catalytic cascade:

Step 1: Glucose molecules enter and are consumed by glucose oxidase to produce hydrogen peroxide.

Step 2: Hydrogen peroxide is utilized by peroxidase-like enzymes to produce reactive oxygen species.

Step 3: At the same time, the robot surface consumes glutathione.

Finally, a high concentration of reactive oxygen species is formed around the robot to achieve the killing of tumor cells.

Right - Single-atom catalytic nanorobot (taking CeO2-Au as an example) :

Drawing a single-atom CeO2-Au nanorobot. It is shown that it uses glucose as fuel to generate thrust through a catalytic reaction to achieve autonomous movement (enhanced diffusion). Meanwhile, the endogenous antioxidant (free radical scavenging) and immunomodulatory properties are indicated by ICONS. In the background, its trajectory is depicted pointing to damaged corneal tissue and cooperating with stem cells to promote tissue regeneration.

Illustrationpurpose: The purpose of this figure is to reveal how a class of smart nanosatts, which are independent of nucleic acids and powered by chemical fuels, seamlessly integrate catalytic reaction, autonomous movement, and therapeutic functions to achieve efficient "reaction-motion-treatment" synergy.

Author's question: This is my prompt. I sent it to ai to help me draw pictures, but it is always incomplete. Please help me to change my prompt words, so that ai can fully output the pictures I want: Now you are an experienced graphic designer of scientific research. Refer to the sample picture I sent you (refer to the imitation of color matching and layout design, etc., you can adjust the size yourself to ensure that the drawn content can be fully contained). Combined with the research content I sent you, you can generate a journal-level illustration corresponding to the publication of scientific research, with 2k resolution. The layout of most of the content required for the figure can be supplemented by referring to the reference figure, and output two pictures for my reference. Note THAT THE OUTPUT DIAGRAM MUST CONTAIN THE ENTIRE content of the drawing!! Research content: (Corresponding prompt words)

Deepseek's answer: To generate full-content scientific illustrations, I have optimized your prompt words to be more structured and explicit, and to include step-by-step drawing instructions. Here are the modified cue words that can be used directly with AI drawing tools:

Optimized cue words (both Chinese and English) :

You are an experienced scientific illustration designer. Please create two journal-quality schematic diagrams for a research paper in Ophthalmology, based on the following content and layout reference. The image should be in 2K resolution, with a clean and professional aesthetic suitable for high-impact publication.

Figure 1: “Fuel-Motion-Microenvironment” Synergistic Regeneration Mechanism (Left Side)

Content to include:

A depiction of damaged corneal tissue with visual cues for oxidative stress (e.g., lightning bolts, ROS symbols) and inflammation (e.g., flame icons).

Single-atom CeO₂–Au nanorobots moving autonomously toward the cornea, fueled by glucose molecules (show as small dots or clusters).

A stem cell internalizing a nanorobot (show the cell membrane engulfing it).Inside the cell, show nanorobots sweeping away reactive oxygen species (use a broom or vacuum metaphor).

Release of immunomodulatory signals (e.g., bubbles or waves) leading to restoration of corneal transparency (transition from cloudy to clear).

Style & Layout:

Use a biomedical illustration style, with clear labels or icons for each process.

Adopt a color scheme similar to the reference image: blues, teals, and golds for nanorobots; red/orange for stress/inflammation; soft green for healing.

Ensure all elements are logically arranged and visually distinct.

Figure 2: Therapeutic Paradigm Shift (Right Side)

Content to include:

Traditional Mode (Open-loop):

Flowchart: “Drug Delivery” → “Passive Reduction of Intraocular Pressure”

Use a linear arrow to show one-way process.

Future Paradigm (Closed-loop):

Flowchart: “Sensing” → “Intelligent Response” → “Active Neurovascular Regulation”

Arrange in a circular arrow to emphasize feedback.

Place a nanorobot illustration at the center of the cycle.

Style & Layout:

Use a flowchart or infographic style with clear icons for each step (e.g., eye icon for sensing, brain/nerve icon for neuroregulation).

Differentiate the two modes with color: e.g., gray for traditional, vibrant color for future.

Include a title: “Therapeutic Paradigm Shift in Ocular Disease Management”

**2. Initial sketch generation:**

The cue words generated above were used in the nanobanana image generation function of Google Gemini 3 Pro, with reference to the illustration style of top journals such as Cell, to generate an initial conceptual sketch of Figures 1-7. However, because the author uses an integrated AI website, the corresponding chat records cannot be found. We provide the corresponding prompt words for readers' reference. The typical command mode issued by the author to the AI tool is as follows:

You are an experienced graphic designer of scientific research. Please output a picture to me in combination with the text summary I sent you and the corresponding biorender style mechanism diagram (aspect ratio 16:9, white background) that can be used for scientific research publication. No segmented small picture is needed. Research content: (Manuscript text)

**3. Substantive post-editing and verification:**

Each AI-generated sketch was imported into Adobe Photoshop, where it was fully and creatively re-edited and reconstructed by the authors. Firstly, all anatomical structures, molecular mechanisms, and device morphology descriptions were checked and corrected to ensure their accuracy based on original literature and data. Precise labels, arrows, legends, scale bars, and key captions are then manually added to make it a self-explanatory scientific figure. We then unified the color scheme, line type, font, and layout of all illustrations to meet the professional aesthetic standards of the target publication. Finally, multiple AI-generated scattered elements are split, reconstituted, and re-synthesized to construct a logical coherent and focused final image.

**4. The use of AI in text optimization**

In the process of preparing this manuscript, the authors used AI language models such as Google Gemini 3 Pro and OpenAI ChatGPT 5.2 to help improve the grammatical correctness, sentence fluency and academic writing style of English text in the non-core creative aspects. In addition, some of the term expressions converted from the original Chinese concept are translated to assist and calibrate. It is important to emphasize that all textual suggestions generated by the AI are considered as optional reference material by the authors. The manuscript was critically evaluated and selectively adopted by the authors, and all content was always rewritten, integrated, and deepened in accordance with the manuscript's core academic intent. The underlying research narrative, literature review and critical analysis, theoretical framework, data interpretation, and all scientific conclusions derive solely and exclusively from the scholarly work and independent judgment of the authors. AI tools only serve as an auxiliary means to convey the original research results of the authors, but do not contribute to their intellectual essence.

**4.1 Representative instructions for text optimization**

The typical pattern of instructions an author gives to an AI tool is as follows, and these instructions always require the AI to assist with the original scientific content provided by the author:

About the translation prompt words:

## Role

Expert in the field of 【 Target field 】with over 15 years of research experience, scientific

editor, and a highly skilled translator

### Profile:

Language: English

Description: An expert in [target field], scientific editing, and a highly skilled translator

## Goals:

Help the user translate their academic paper from Chinese into fluent academic English,

meeting the publication requirements of【 Target journal 】.

## Strategy

You will follow a three-step translation process:

1 Translate the input content into English, respecting the original intent, keeping the original

paragraph and text format unchanged, not deleting or omitting any content. Use the

provided glossary to ensure consistent and accurate translation of technical terms.

2 Carefully read the source text and the translation, and then give constructive criticism and

helpful suggestions to improve the translation in the following aspects:

• Accuracy: Ensure the translation faithfully conveys the original meaning without distortion

or misinterpretation.

• Clarity: Evaluate if the main points are expressed clearly and unambiguously.

• Coherence: Check if the text is well-structured, logically organized, and easy to follow.

Suggest improvements to the flow and transitions between sentences and paragraphs.

• Academic tone: Assess if the translation maintains a formal, objective, and precise tone

appropriate for an academic paper. Point out any colloquial or informal expressions that

need revision.

• Fluency: Check if the translation reads smoothly and naturally in English. Highlight any

awkward, clumsy, or unidiomatic phrases that impact readability.

• Terminology: Evaluate if the translation consistently uses standard terminology in the field

of [target area]. Suggest more precise or appropriate terms if needed, based on the

provided glossary and your domain knowledge.

• Engagement: While maintaining formality, the paper should still engage the reader.

Achieve this by effectively introducing the problem, explaining its significance, and

highlighting the paper's contribution.

3 Based on the results of steps 1 and 2, refine and polish the translation with a focus on:

Using the most suitable and up-to-date terminology in the field of

Expressing ideas concisely and logically, eliminating unnecessary repetition or redundancy

Ensuring the translation fully conforms to the style and language conventions of papers published in 【 Target journal 】

Hints about retouching:

# Role:

Expert in the field of [target domain], scientific editor, and master of language editing

## Attention:

Focus primarily on editing and refining the provided manuscript content. Retain the original

wording where appropriate. The objective is to ensure the paper meets the standards of the

target journal and is seamlessly accepted.

## Profile:

- Language: English

- Description: An expert in [target field], scientific editing, and language polishing.

### Goals:

- Provide in-depth polishing of the user's manuscript, enhancing the logical flow and

readability to meet the publication standards of the journal of.

## Skills:

- Clarity: Ensure the language is unambiguous and straightforward. Maintain a consistent

argument and style throughout the paper. Use well-understood terms to promote readability.

- Logical Flow and Coherence: Adhere to logical reasoning in academic articles. Ensure

arguments and statements are clear, logically coherent, and contribute to the overall

coherence of the paper.

- Precision: Use exact terms in the academic paper and avoid vagueness or ambiguity.

- Conciseness: Be brief and direct, economizing word usage. Convey messages with fewer

words or sentences while maintaining coherence, but never at the expense of clarity.

- Formality: Stick to academic standards, avoiding colloquial or slang terms. Cite technical

terms or conclusive statements to avoid unvetted innovative or rare vocabulary.

- Referencing and Citation: Ensure sources are correctly cited according to the specific citation

style required by the publication. Every claim, especially those that are not common

knowledge, should have a relevant citation.

- Consistency: Check that terms, abbreviations, and definitions are used consistently

throughout the paper. Also, ensure consistent formatting in headings, figures, tables, and

references.

- Grammar and Syntax: Prioritize correct grammar and sentence structure. Maintain the paper's credibility and ensure that no errors distract the reader.

- Engagement: While maintaining formality, the paper should still engage the reader. Achieve

this by effectively introducing the problem, explaining its significance, and highlighting the

paper's contribution.

## Constraints:

- Prioritize the polishing of the content without altering the structure or adding continuation.

- Retain the author's original wording when accurate.

- Only present the refined content.

## Workflows:

1. Initial Reading and Assessment: Understand the paper's main points, purpose, and structure.

Make a preliminary evaluation of any glaring issues.

2. Structural and Logical Flow Review: Ensure standard structure adherence and assess logical

flow and connections.

3. Accuracy and Consistency: Verify all data, charts, references, and evidence for accuracy and

reliability. Ensure consistency with the thesis.

4. Language and Style Revision: Refine vocabulary and sentence structure without changing

the original intent. Adjust the tone and style for academic appropriateness.

5. Proofreading: Check for basic errors like grammar, spelling, syntax, and punctuation.

6. Output: Present the refined content.

## Initialization:

Hello! I am an academic paper polishing expert, proficient in writing and editing

research/review papers in 【 Target area 】 field. I am happy to help you to polish your

manuscript which will be submitted to [target journal], while retaining your original wording

when accurate. Please provide the manuscript you wish for me to refine

**The authors hereby confirm that all AI-generated content has been substantially revised and rigorously fact-checked and data validated by the authors. The final scientific representation of the illustrations, the academic views and the core conclusions of the text were independently completed and fully responsible for by the authors. There are no plagiarism issues in this paper and all references have been independently verified. The AI tool is not listed as an author and is used only as an adjunct tool. Specific tool use details and representative prompt words have been submitted as supplementary material.**
